# Acknowledgment to Reviewers of *Plants* in 2020

**DOI:** 10.3390/plants10020226

**Published:** 2021-01-25

**Authors:** Plants Editorial Office

**Affiliations:** MDPI AG, St. Alban-Anlage 66, 4052 Basel, Switzerland

Peer review is the driving force of journal development, and reviewers are gatekeepers who ensure that *Plants* maintains its standards for the high quality of its published papers. Thanks to the cooperation of our reviewers, in 2020, the median time to first decision was 14 days and the median time to publication was 35 days. The editors would like to express their sincere gratitude to the following reviewers for their precious time and dedication, regardless of whether the papers were finally published:
Abdelrahman, MostafaLi, ZhongyiAbedi, TayebehLi, ZhouAbinaya, ManivannanLiagre, BertrandAbrahamczyk, StefanLiakos, IoannisAbrieu, ArianeLiang, ZhikaiAbrisqueta, IsabelLiao, YuxingAbugri, Daniel A.Liberati, DarioAcharjee, AnimeshLibik-Konieczny, MartaAcosta Motos, Jose RamónLibutti, AngelaAcosta-Jurado, SebastiánLichocka, MałgorzataAdachi, ShunsukeLico, ChiaraÁdám, Attila L.Lidon, FernandoAdamakis, Ioannis-DimosthenisLiede-Schumann, SigridAdamec, LubomírLieffers, VictorAdamidis, George C.Ligaba-Osena, AyalewAdamidis, GeorgiosLigrone, RobertoAdamowski, MaciekLiguori, GiorgiaAdams, JamesLijavetzky, DiegoAdegbola, RaphaelLim, SangbinAdhikari, KedarLima-Brito, JoséAdhikari, TikaLimpens, ErikAfonnikov, Dimitry ArkadievichLin, ChaoyuanAfonso, Andrea Luísa FernandesLin, Choun-seaAgnieszka, SzopaLin, FanÅgren, Göran I.Lin, Hai-ShuAgrimi, GennaroLin, HuaiyingAguiar, CristinaLin, Kuan-HungAguiar, Francisca C.Lin, Shian-RenAgustí-Brisach, CarlosLin, Ying-HongAhmad, MargaretLindberg, SylviaAhmad, WaqarLing, ChenAhmed, MukhtarLingua, GuidoAhn, Mi-JeongLinkies, AdaAiello, DaliaLiobikas, JuliusAires, AlfredoLionetti, VincenzoAizawa, MineakiLiovic, IvicaAkhgari, AmirLipińska, HalinaAkkak, AzizLips, KatrinAksmann, AnnaLira-Medeiros, CatarinaAlabdullah, Abdul KaderLira-Saade, RafaelAlam, MobashwerLisec, JanAlamillo, Josefa MuñozLisek, JerzyAlba, ChristinaLitwin, Ireneusz BernardAlbergamo, AmbroginaLiu, ChangningAlbert, RekaLiu, FulaiAlboresi, AlessandroLiu, GuoxiangAlejandro, SantiagoLiu, Hai-yangAlexandrov, Oleg S.Liu, KeAlferez, FernandoLiu, KunhsiangAli, AkhtarLiu, LeiAli, AnwarLiu, LiangliangAli, Muhammad MuddassirLiu, LijunAlicja, Macko-PodgorniLiu, Shang-Yin VansonAliki, XanthopoulouLiu, ShaoyangAlina, WiszniewskaLiu, UdayanganiAlison, SmithLiu, WeiAlizadeh, HosseinLiu, XiaoxiAlkowni, RaedLiu, YongliangAllakhverdiev, SuleymanLiu, YongqiangAllegrezza, MarinaLiu, ZhaohuiAllen, Edith B.Liu, ZijuanAllen, Tom W.Livrea, Maria AntoniaAlonso-Prados, José LLlorens, EugenioAlptekin, BurcuLlugany, MercèAlshaal, TarekLo Bianco, RiccardoÁlvarez Fernández, InésLo Piero, Angela RobertaAlvarez, Juan B.Lobiuc, AndreiÁlvarez-Suarez, José M.Lobón, Natividad ChavesAlves, MaraLocascio, AntonellaAlzubaidi, LaithLochmanová, GabrielaAmarowicz, RyszardLoeza Alcocer, Jose EmanuelAmato, MarianaLogacheva, MariaAmbrožič-Dolinšek, JanaLogozzo, GiuseppinaAmeye, MaartenLohwasser, UlrikeAmillis, SotirisLoiko, Sergey VAmini, HajarLomonaco, TommasoAmirkhani, MasoumeLondon, LucilleAmoo, Stephen OluwaseunLopes, Everaldo AntônioAmoriello, TizianaLopes, GracilianaAmorós, AsunciónLópez, Mercedes G.Amsbury, SamLopez-Arredondo, DamarAna, Fernández-OcañaLopez-Marin, JosefaAnai, ToyoakiLopez-Molina, LuisAncín, MaríaLora, JorgeAncona-Contreras, VeronicaLord, EtienneAncuceanu, RobertLord, JaniceAndelkovic, IvanLorenz, PeterAnderson, AnneLorenzis, Gabriella DeAndrade Cetto, AdolfoLoreti, ElenaAndrade, Eugénia DeLorite, MariaAndreas, BühlmannLosapio, GianalbertoAndreason, SharonLoskutov, IgorAndrei, LobiucŁotocka, BarbaraAndrei, SandaLou, YonggenAndres, Maria FeLoveridge, JoelAndresen, Christian G.Lu, Pei-LuenAndrew, TedderLu, YouAndroniki (Nina), PapafilippakiLubell, JessicaAndrosiuk, PiotrLubkowski, KrzysztofAneva, Ina YosifovaLucarini, MassimoAngeles-Shim, Rosalyn B.Luccini, LuigiAngelini, PaolaLucena, CarlosAngelino, DonatoLucero, Leandro ExequielAngiolini, ClaudiaLucioli, SimonaAnisimova, IrinaLücking, RobertAnken, ThomasLudidi, NdikoAntal, DianaLuigi, PalmieriAntoch, JaromirLukina, NataliaAntonets, KirillLukinac Čačić, JasminaAntonio Morrone, LuigiLukomska-Szymanska, MonikaAntonio Speretto, RaulLukova, PaolinaAntoniou, ChrystallaŁukowski, AdrianAntonkiewicz, JacekLullien, ValerieAntonny, BrunoLumini, EricaAntony, JaneLuo, XubiaoAntunes, DulceLupini, AntonioAnver, ShajahanLuque, FranciscoAoki, KohLushington, GerryAparicio, FredericLüthje, SabineAparicio, Pedro MLüttge, Ulrich E.Appendino, GiovanniLuvisi, AndreaAprile, AlessioLux, AlexanderAprodu, IulianaLux, ThomasAraki, RyoichiŁyczakowski, JanArancibia, MirariŁyczko, JacekAranda, XavierM. Barton, AndrewAraniti, FabrizioMa, GuojiaArasimowicz-Jelonek, MagdalenaMa, SiyanAraújo, Alberto N.Ma, WeiAraújo, SusanaMa, XiaochiAravanopoulos, FilipposMa, ZhengqiangArbona, VicentMaag, DanielArc, ErwannMaalouf, Fouad S.Arcone, RosariaMachado, Maria De Fátima P.S.Aremu, Adeyemi OladapoMachida-Hirano, RyokoArencibia, Ariel DomingoMachkour-M’Rabe, SalimaArévalo, José RamónMacía, Dr. ManuelArgentieri, Maria PiaMacQueen, Alice H.Argento, SergioMaděra, PetrArgumedo-Delira, RosalbaMaekawa, ShugoArie, TsutomuMaggi, FilippoAriel, FedericoMaggini, RitaArita, MasanoriMaggio, AlbinoAriz, IdoiaMaghuly, FatemehArmstead, IanMagiera, AnjaArnaiz, AnaMagnano Di San Lio, GaetanoArnao, Marino BañónMagnard, Jean LouisArnaud, NicolasMagrini, SaraAronen, TuijaMaguilla, EnriqueArpaia, SalvatoreMagyar, ZoltánArriagada, CésarMahato, NeelimaArru, LauraMaioli, MargheritaArtemyeva, AnnaMaiuolo, JessicaArtyszak, ArkadiuszMajeský, ĽubošAryal, RishiMajewska, MałgorzataAsano, KazuhitoMajor, Ian T.Ashour, Mohamed LotfyMajsztrik, JohnAsins, M.J.Makarevitch, IrinaAsnicar, FrancescoMakino, AmaneAsquith, Christopher R. M.Maksimov, Igor V.Astier, JeremyMalandrino, MeryAtamian, HagopMalatesta, LucaAthanasios, DalakourasMalatinszky, ÁkosAttila, FehérMaleita, CarlaAubry, SylvainMalfeito-Ferreira, ManuelAudergon, Jean MarcMaliga, PalAugustine, Sruthy MariaMalinowski, RobertAvato, PinarosaMalisch, Carsten S.Avellan, AstridMalkowski, EugeniuszAvramidou, EvangeliaMalla, SubasAvramova, ViktoriyaMalladi, AnishAvtzis, Dimitrios N.Mallor, CristinaAwad, MohamadMalusà, EligioAwasthi, MayankaMamolos, AndreasAyala-Zavala, J. F.Mancianti, FrancescaAyaydin, FerhanMancinelli, RobertoAzad, Obyedul KalamMancini, InesAzarin, Kirill VitalievichMandal, Mihir KumarAzinheira, Helena G.Mandzhieva, Saglara S.Azuma, WakanaMang, Stefania MirelaB. Santos, RitaManivannan, AbinayaBabojelić, Martina SkendrovićMankin, Richard W.Baccelli, IvanMannucci, CarmenBachmair, AndreasMano, ShojiBackhouse, DavidMano, YoshiroBackor, MartinMantzoukas, SpiridonBaczewska-Dąbrowska, Aneta HelenaManuel Perez-Perez, JoseBączkiewicz, AlinaMarahatta, SharadchandraBaczyńska, DagmaraMarasek-Ciolakowska, AgnieszkaBadagliacca, GiuseppeMaraseni, TekBadea, AnaMarć, Małgorzata AnnaBadea, MihaelaMarček, TihanaBader, MaaikeMarchand, ChristopheBadiani, MaurizioMarchand, GenevièveBadjakov, IlianMarchand, Patrice A.Badr, AbdelfattahMarchetti, MartaBaek, Kwan-hyumMarciniak, KatarzynaBaez, Selene BaezMarcińska, IzabelaBagchi, RammyaniMarco Montori, PedroBagniewska-Zadworna, AgnieszkaMarco, FranciscoBahcevandziev, KirilMarczak, MałgorzataBahmani, RaminMarek, Michal V.Bai, BingMaresca, MarcBai, Geng(Frank)Maresca, VivianaBai, RenrenMargaoan, RodicaBaig, Mohammad HassanMargis, RogerioBailey, BryanMargońska, Hanna B.Bailly, ChristopheMarguerit, ElisaBajaj, RuchikaMaria Musarella, CarmeloBajer, TomasMarilyn, WarburtonBajgain, PrabinMarincs, FerencBajwa, Ali AhsanMarino, BrunoBajwa, Vikramjit S.Marion-Poll, AnnieBakkestuen, VegarMarković, MonikaBalasubramanian, BalamuralikrishnanMarkowicz-Piasecka, MagdalenaBalažová, AndreaMarla, SandeepBalázs, VargaMarler, ThomasBaldauf, Jutta A.Marmiroli, MartaBaldermann, SusanneMarmiroli, NelsonBaldini, MarioMarques, IsabelBaldoni, ElenaMarques, NatáliaBalestrini, RaffaellaMarrelli, MariangelaBaležentienė, LigitaMarshall, Michael W.Balfagón Sanmartín, DamiánMarsol, AlexisBalick, Michael J.Marsolais, FredericBallen Taborda, CarolinaMárta, LadányiBallesteros, DanielMartelli, GiuseppeBallottari, MatteoMartellos, StefanoBalslev, HenrikMartí, ClaraBalusamy, Sri RenukadeviMartin, Jonathan G.Baluška, FrantišekMartina, MatteoBalzano, AngelaMartínez Ávila, Guillermo Cristian GuadalupeBan, YusukeMartinez De Toda, FernandoBanach, ArturMartínez Vázquez, Maria LuisaBanasiak, AlicjaMartínez, Enrique A.Bandaralage, Jayeni Chathurika Amarathunga HitiMartínez, SidoniaBandurska, HannaMartínez-Álvarez, FranciscoBanerjee, AparajitaMartínez-Gómez, PedroBanilas, GeorgiosMartínez-Hernández, FabiánBanks, Jonathan M.Martínez-Téllez, Miguel ÁngelBao, YongpingMartín-Gil, JesusBarabasz-Krasny, BeataMartinka, MichalBaránek, MiroslavMartinoia, EnricoBaranova, EkaterinaMartins, M RosarioBarba, Francisco J.Martins, NataliaBarba-González, RodrigoMartins-Loução, Maria AméliaBarbato, RobertoMartos, VanessaBarberis, AntonioMasatake, KanaiBarcelo, JuanMaserti, Bianca ElenaBardají, EduardMashima, RyuichiBargmann, BasMasi, AntonioBari, EliaMasi, MarcoBarłóg, PrzemysławMasiero, SimonaBarna, BalazsMasin, RobertaBarnes, PaulMašková, TerezaBarone, EttoreMassa, DanieleBarrajon, EnriqueMassai, RossanoBarreca, DavideMassimino Cocuzza, GiuseppeBarrios-Masias, Felipe H.Massouras, TheofylaktosBarros, AnaMastanjević, KristinaBarros, Marcelo PaesMastinu, AndreaBarros, Pedro M.Mastrangelo, Anna M.Barsanti, LauraMastrorilli, MarcelloBarta, CsengeleMasuda, TatsuruBartelheimer, MaikMata-Pérez, CapillaBartlow, Andrew W.Mateo-Bonmatí, EduardoBartolini, SusannaMaterska, MałgorzataBartoš, MichaelMatha, Shivaraj SheelavantaBartucca, Maria LuceMathesius, UlrikeBascom, Carlisle S.Mathieu-Delalande, MagalieBasheer, JasimMatilla, Angel J.Bashir, Muhammad AjmalMatilla, MiguelBasile, AdrianaMatkowski, AdamBaskin, Carol C.Matłok, NataliaBaskin, TobiasMatoba, NobuyukiBaslam, MarouaneMatos, ManuelaBasnet, Bhoja RajMatoša Kočar, MajaBassi, DavideMatraszek-Gawron, RenataBastida, FernandoMatsubara, KazukiBasu, SupratimMatsuda, FumioBates, PhilipMatsumoto, TakahiroBatley, JacquelineMatsushima, RyoBattino, MaurizioMatsuzaki, KeiichiBauer, NatašaMattana, MonicaBaum, MichaelMattei, BenedettaBazan, GiuseppeMatthew, BiwerBazihizina, NadiaMatthews, Benjamin J.Bazile, DidierMatthysse, Ann G.Bazos, IoannisMattoo, AutarBazylko, AgnieszkaMattoscio, DomenicoBeamish, AlisonMatula, RadimBeccari, GiovanniMatusikova, IldikoBecker, YvonneMatveeva, TatianaBeckie, Hugh J.Mauro, Rosario PaoloBednarek, PiotrMauromicale, GiovanniBedon, FrankMayton, HilaryBedre, ReneshMazourek, MichaelBegum, KohinoorMazur, MajaBehm, Carolyn A.McAusland, LornaBehr, MarcMcCarthy, ElizabethBeilby, MaryMcCormick, AlistairBelbahri, LassaadMcCubbin, AndrewBelda-Palazón, BorjaMcFarlane, Heather E.Belka, MartaMcFeeters, RobertBell, LukeMcintosh, RobertBellairs, SeanMcKenzie-Gopsill, AndrewBellaloui, NacerMcKeown, Peter C.Bellot, María José GómezMcNab, HenryBellucci, MicolMcNeely, Jeffrey A.Benarba, BachirMcPhee, KevinBen-Arie, RuthMedeiros, Juliana SBenavides-Mendoza, AdalbertoMedina Juarez, Luis AngelBencivenga, StefanoMedina, Francisco JavierBendokas, VidmantasMedo, JurajBenech-Arnold, Roberto LuisMeeks, CalvinBenedec, DanielaMehariya, SanjeetBenedek, CsillaMeland, MekjellBenelli, CarlaMelendez-Martinez, AntonioBeng Kah, SongMeletiou-Christou, Maria-SoniaBeni, ClaudioMelillo, Maria TeresaBenitez-Alfonso, YoselinMellano, Maria GabriellaBennett, ALAN B.Mempel, HeikeBenny, JubinaMenassa, RimaBenoit, MatthiasMencherini, TeresaBenrey, BettyMenegazzi, MartaBento, José Maurício SimõesMENEGUZZO, FRANCESCOBerestetskiy, AlexanderMengiste, TesfayeBerg, TrygveMenkissoglu-Spiroudi, UraniaBerger, BeatriceMennucci, BenedettaBergler, HelmutMensuali, AnnaBergman, ChristineMentana, AnnalisaBergmeier, ErwinMenze, MichaelBergougnoux, VéroniqueMenzel, Christopher M.Berisso, KebedeMerah, OthmaneBeritognolo, IsaccoMerchante, CatharinaBerkowitz, GeraldMerfield, CharlesBerlanga, Ángel MéridaMerganičová, KatarínaBerna, ÀngelMerino, IreneBernacchia, GiovanniMerret, RémyBernal, Freddy AlexanderMertlíková-Kaiserová, HelenaBernard, Ernest C.Messyasz, BeataBerne, SabinaMesterhazy, AkosBerni, RobertoMeulia, TeaBernier, LouisMewis, IngaBernstein, NiritMeyer, ChristianBerrocal-Lobo, MartaMezzetti, BrunoBerruyer, RomainM-Hamvas, MártaBertea, CinziaMiazzi, Monica MarilenaBertin, SabrinaMiceli, AlessandroBertini, LauraMiceli, FabianoBertrand, BenoîtMiceli, NataliziaBervillé, AndréMichaeli, SimonBešlo, DragoMichalak, MarcinBesnard, GuillaumeMichalet, RichardBessudo, CathyMichard, ErwanBetekhtin, AlexanderMichel, PiotrBetti, CamillaMichel, SebastianBetti, MarcoMichele, RobertoBhati, Kaushal KumarMicillo, RaffaellaBhattacharjee, SonaliMidmore, DavidBhattacharya, SamikMierek-Adamska, AgnieszkaBhattarai, KrishnaMihali, CristinaBhattarai, SurendraMihaylova, DashaBhusal, NarayanMihovilović, Anita BošnjakBiagi, MarcoMikami, KojiBiałowiec, AndrzejMikuła, AnnaBian, ZhonghuaMikulic-Petkovsek, MajaBianco, ArmandodorianoMilan, MarcoBianco, CarmelinaMilc, JustynaBianco, RiccardoMildažienė, VidaBiancolillo, AlessandraMilella, LuigiBidel, L.P.R.Milham, PaulBiedermann, DavidMilhinhos, AnaBiel, WiolettaMiličević, TihomirBiernasiuk, AnnaMillar, AnthonyBiesiada, AnitaMiller, W. AllenBigelow, Seth W.Millet, Emilie JBilodeau, GuillaumeMilner, JoelBilska-Kos, AnnaMilojković-Opsenica, Dušanka M.Biondi, StefaniaMimica-Dukic, NedaBipfubusa, MarieMin, Won LeeBirsa, Mihail LucianMinassi, AlbertoBiselli, ChiaraMinganti, VincenzoBishopp, AnthonyMinicka, JuliaBisht, VikramMinissale, PietroBiswas, Manosh KumarMino, MasanobuBizimungu, BenoîtMinto, Robert E.Blaby-Haas, Crysten E.Mir, Tahir MaqboolBlagodatski, ArtemMira, SaraBlanco, AntonioMiranda, AnaBlanco-Pastor, José L.Miranda-Apodaca, JonBlanco-Salas, JoséMirás-Avalos, José ManuelBlando, FedericaMirmazloum, ImanBłaszczyk, LidiaMishev, KirilBlazevic, IvicaMishra, Amit KumarBlicharski, TomaszMishra, AwdheshBlock, AnnaMishra, DivyaBlomme, JonasMishra, PriyankaBlouin, ArnaudMita, GiovanniBoari, FrancescaMitaine-Offer, Anne-ClaireBoba, AleksandraMitrović, MiroslavaBobev, SvetoslavMitsui, YukiBoccacci, PaoloMitsuya, ShiroBocianowski, JanMitsuyama, SusumuBock, CliveMiyamoto, KojiBoczkowska, MajaMiyazawa, ShinichiBogdanov, Alexey M.Mizzotti, ChiaraBogdanova, Vera S.Mleczek, MiroslawBogdanove, AdamMlinarić, SelmaBogdányi, Franciska TóthnéMo, YoungjunBoisivon, Helene RobertMoccia, StefaniaBojić, MirzaMočibob, MarkoBojinov, BojinMoffat, Caroline S.Bojko, AgnieszkaMohamed, Azza H.Bojko, JamieMohanta, Tapan KumarBolarić, SnježanaMoise, EricBölcskei, HedvigMolesini, BarbaraBoldizsár, ImreMolina Salinas, Gloria MaríaBolibok-Brągoszewska, HannaMolina-Heredia, Fernando P.Bolívar, JorgeMolinier, JeanBölter, BettinaMøller, BirgerBonamore, AlessandraMöller, MichaelBonaterra, AnnaMolnár, AtillaBonciu, ElenaMolony, BrettBongers, FransMonaco, Thomas A.Bonhomme, VincentMoncada, AlessandraBonvicini, FrancescaMondello, VincenzoBorek, SławomirMonfils, Anna K.Borges, Andrés AntonioMonokrousos, NikolaosBorges, RicardoMontagnoli, AntonioBoris, PejinMontalbán, Itziar A.Boros-Lajszner, EdytaMontana, Angel M.Borowiak, Klaudia-AnnaMonte, EnriqueBorrelli, Grazia MariaMonteiro, CarlosBörve, JorunnMonteiro, FilipaBorza, TudorMontenegro, Juan D.Boscaiu, MonicaMontes, NuriaBoscari, AlexandreMontesano, DomenicoBoscaro, ValentinaMontiel, JesúsBoss, PaulMontone, Carmela MariaBosso, LucianoMoolhuijzen, Paula M.Bostan, HamedMoon, Il SooBotez, ElisabetaMoon, KyungBotu, MihaiMoon, SunokBoualem, AdnaneMopper, SusanBouranis, Dimitris L.Mora, FreddyBourbousse, ClaraMorabito, DomenicoBourland, FredMorales, JorgeBoutilier, KimMorales, LauraBovet, LucienMorales-González, José AntonioBowden, LauraMoravcikova, JanaBowman, DarylMorcillo Parra, María ÁngelesBoyce, RichardMoreira, José Claudio FonsecaBozena, SeraMorello, LauraBraca, AlessandraMoreno, JordiBracchi, Valentina A.Mori, KazuhiroBracco, EnricoMoriguchi, TakayaBrader, GünterMorita, KyojiBramanti, EmiliaMorita, ShigetoBranca, FerdinandoMorohashi, KengoBranco-Vieira, MoniqueMorris, Craig F.Brandt, KirstenMorris, Paul FrancisBranham, Sandra ElaineMorrow, JenniferBrankov, MilanMorsy, MustafaBraukmann, Thomas W. A.Mort, AndrewBrazaitytė, AušraMoschou, Panagiotis NikolaouBren, UrbanMoskalev, AlexeyBrennan, Adrian C.Moss, BritneyBrennan, MareeMossel, EricBresta, PanagiotaMostowska, AgnieszkaBrestic, MarianMota Poveda, Juan FranciscoBretfeld, MarioMota, ManuelBrewin, NickMottaghipisheh, JavedBreygina, Maria AMOTTI, RICCARDOBrierley, JennieMourtzinos, IoannisBrisson, JacquesMożdżeń, KatarzynaBrito, CátiaMravec, JozefBrkanac, Sandra RadicMroczek, AgnieszkaBrkljača, MiaMucalo, AnaBroadhurst, LindaMuccilli, VeraBroberg, MartinMucha, JoannaBrogden, KimMuhie, SeidBrokaw, NicholasMühlenhoff, UlrichBronisz, KarolMuhovski, YordanBrumelis, GuntisMule, PaoloBrummell, DavidMulo, PaulaBrumos, JavierMun, Bong GyuBrunetti, CeciliaMundy, D.C.Brunkard, Jacob O.Munir, Tariq M.Bruno, LauraMuñoz, AlfonsoBrychkova, GalinaMuñoz, Jesúsbrzoska, malgorzata michalinaMuñoz-Mingarro, DoloresBubici, GiovanniMünstedt, KarstenBubola, MarijanMuntean, EdwardBuchet, ReneMurata, HitoshiBuchner, PeterMurata, JunBuda, ValentinaMurato, MiguelBueno, MilagrosMurphy, BrianBueso, EduardoMuschietti, JorgeBuijs, GondaMusić, Martina ŠerugaBuitink, JuliaMustafa, Ahmed M.Bujak, TomaszMuszyńska, EwaBujdoso, GezaMutiga, Samuel K.Bulli, PeterMylona, PhotiniBunce, JamesMyrta, ArbenBunch, HeeyounMyśków, BeataBunea, AndreaNa, Chae-InBunea, Claudiu IoanNachappa, PunyaBurch-Smith, TessaNadarajan, JayanthiBurducea, MarianNadeem, MuhammadBurgess, Alexandra JacquelynNadiminti, Pavani PraveenBurgos, LorenzoNagahage, IsuraBurke, Susan J.Nagaki, KiyotakaBurketova, LenkaNagarajan, RagupathiBurkey, KentNagashima, YukihiroBurton, Amy L.Nagel, RaimundBus, Vincent G.M.Nagl, NevenaBusby, Ryan R.Nagy, IstvanBush, Daniel R.Naing, Aung HtayBush, Stephen JNair, Prakash M GopalakrishnanBussmann, Rainer W.Najda, AgnieszkaButardo, VitoNakabayashi, KazumiButnariu, MonicaNakagawa, KouichiCabeza, Ricardo A.Nakamichi, NorihitoCacciola, FrancescoNakamura, TakahiroCacciola, Santa OlgaNakano, MichiharuCaddeo, CarlaNakata, MiyukiCaeiro, Maria FilomenaNalam, VamsiCafasso, DonataNam, Jin-WuCagliero, Cecilia LuciaNambeesan, SavithriCahoon, A. BruceNanasato, YoshihikoCai, GiampieroNanjappa, DeepakCai, WenlongNaoumkina, MarinaCaixeta De Oliveira, HalleyNapoli, Edoardo MarcoCala, AntonioNapoli, MarcoCalderini, OrnellaNaranjo, TomásCalha, IsabelNarbona, EduardoCallis, JudyNarciso, Joan OñateCALZARANO, FrancescoNardini, AndreaCamacho, MaríaNarouei-Khandan, Hossein A.Camacho-Cristóbal, Juan J.Nassini, RominaCamargo Rodríguez, Anyela ValentinaNatarajan, PurushothamanCamele, IppolitoNavar, JoséCamiña García, MercedesNavarro, JoaquinCamino, CarlosNavarro-León, EloyCampbell, MichaelNawade, BhagwatCampilho, AnaNawaz, FahimCampisi, PatriziaNawaz, Muhammad AmjadCampos, DeboraNawirska-Olszańska, AgnieszkaCampos, ReinaldoNawracała, JerzyCanales, JavierNawrot-Hadzik, IzabelaCanas, RicardoNazar, Ross N.Candela, HéctorNaznin, Most TaheraCandido, VincenzoNebeská, DianaCanedoli, ClaudiaNecas, TomasCaneva, GiuliaNegri, GiuseppinaCanhoto, Jorge M.Nehela, YasserCanini, AntonellaNeilson, RoyCánovas, FranciscoNeji, MohamedCantamessa, SimoneNelson, DavidCantini, ClaudioNelson, Jessica M.Cantos, ManuelNemenyi, AndrasCanut, HerveNemes, KatalinCao, ZheNemeskéri, EszterCaparrotta, StefaniaNémeth, András GyörgyCapasso, RaffaeleNémeth, Zsolt IstvánCaplan, AllanNeng, NunoCaputo, LuciaNeri, DavideCaraballo-Rodríguez, Andrés MauricioNerva, LucaCaracciolo, GiuseppinaNeta Gostin, IrinaCárdenas Pérez, StefanyNeufeld, HowardCardinale, MassimilianoNewcombe, GeorgeCardoso, HéliaNewman, Steven E.Cardoso, SusanaNg, Carl K. Y.Caretto, SofiaNgo, Anh H.Carillo, PetroniaNicolescu, Valeriu-NorocelCarluccio, Anna VittoriaNicoletti, RosarioCarman, John G.Nicolia, AlessandroCarneros, ElenaNicosia, AldoČarni, AndražNiedbała, GniewkoCarnicelli, VeronicaNieddu, GiovanniCaro, ElenaNiedojadło, JanuszCarocho, Márcio SoaresNiehaus, Thomas DCarradori, SimoneNielsen, BirtheCarral Vilariño, Emilio V.Nielsen, BrentCarranca, CorinaNiemann, JanettaCarrasco, PedroNiemczak, MichalCarrasquinho, IsabelNieto, GemaCarrière, YvesNiewiadomska, EwaCarriquí, MarcNigro, FrancoCarro, LorenaNikoloudakis, NikolaosCartmill, Andrew D.Nikolova, Petia SimeonovaCartmill, Donita L.Nilsen, Erik TCarullo, GabrieleNin, StefaniaCaruso, GianlucaNina, BrutchCaruso, MarcoNing, XuhuiCarvajal, MicaelaNishio, SogoCarvalho, Ana Maria PintoNishiyama, MakotoCarvalho, LuísaNita, AndreeaCasanova-Sáez, RubénNita, MizuhoCasas, Ana M.Niu, FurongCasas, José LuisNizhnikov, Anton A.Casciaro, BrunoNogales, AmaiaCaser, MatteoNoguchi, KoCaseys, CelineNoguera Artiaga, LuisCasino, PatriciaNoh, Yoo-SunCasoni, DorinaNonomura, Ken-ichiCassab, Gladys I.Nosalewicz, ArturCastellano, GloriaNosek, MichałCastellanos, AlejandroNosov, AlexanderCastiglione, Monica RuffiniNotarbartolo, MonicaCastiglione, StefanoNoulas, ChristosCastilho, Paula C.Novello, VittorinoCastillo, AnaNovikov, AndriyCastillo, JesúsNovikov, Arthur I.Castillo, PabloNovo, LuísCastillo-Gonzalez, Claudia MarcelaNowack, Moritz K.Castro, Pedro HumbertoNowak, MichałCastroverde, Christian Danve M.Nowakowska, JustynaCasucci, CristianoNowicka, AnnaCatalá, MyriamNowicki, MarcinCataldo, SalvatoreNtalli, Nikoletta G.Catarino, LuisNuc, PrzemysławCautela, DomenicoNunez, Gerardo H.Cavalaris, ChrisNuortila, CarolinCavallaro, ValeriaNussbaumer, ThomasCeccarelli, MarilenaNutricati, ElianaCecchi, LorenzoNuttall, Patricia AČegan, RadimNyine, MosesĆelepirović, NevenkaO’Hanlon, RichardCelesti, MarcoO’Keeffe, Kayleigh RCeliński, KonradO’Leary, Brendan M.Cellini, AntonioO’Neil, GregoryCembrowska-Lech, DanutaOancea, FlorinČepulienė, RitaOberemok, Volodymyr V.Cerdà, ArtemiObulisamy, Parthiba KarthikeyanČerevková, AndreaOda, AtsushiČernáková, LuciaOddo, ElisabettaČerný, MartinOgata, YoshiyukiCerri, MartinaOgawa, TakumiCervantes, EmilioOgden, Kimberly L.Cesbron, SophieOghenekaro, Abbot O.Česonienė, LaimaOgórek, RafałCessna, StephenOgris, NikicaCetera, PaolaOgrodowicz, PiotrChagne, DavidOgunjirin, AdebowaleChaki, MouniraOh, JaehyeonChan, Yul YooOh, Man-hoChandrasekaran, MurugesanOhnishi, KouheiChang, Fang-RongOhshima, KazusatoChang, Hung-ChiOhyama, TakujiChang, JiyulOkamoto, TakashiChang, Pearl ChunOklestkova, JanaChang, Yuan-YenOkoń, SylwiaChao, Louis KuopingOkorski, AdamChapman, MarkOláh, ViktorChappell, Thomas M.Olaru, Octavian TudorelChardon, FabienOlea, Andres F.Chardot, ThierryOlech, MartaChase, ChristineOlejar, Kenneth J.Chateigne-Boutin, Anne-LaureOleszek, WieslawChatterton, SyamaOlga, GavrilovaChatzistathis, TheocharisOliveira, Antonio Costa DeChaudhary, Dhiraj KumarOliveira, Rui S.Chawade, AakashOlmos, AntonioChe, Chun-TaoOlszewska, Monika ACheema, JitenderOmar, Ahmad A.Cheimona, NikolinaOmelchenko, Denis O.Chekanov, KonstantinOndreičková, KatarínaChen, Chang-ChangOndřej, VladanChen, Grace QOng, Eng ShiChen, HuiOniga, IlioaraChen, Jen-TsungOniszczuk, AnnaChen, JinyiOnodi, GaborChen, Peng-WenOono, RyokoChen, WenOono, YutakaChen, YinglongOppermann, MarkusChénais, BenoitOppert, BrendaCheng, Juei-tangOprea, ElizaCheng, QunkangOpricǎ, LăcrămioaraCheng, Yuan-BinOracz, KrystynaCheon, Choong-illOrdas, BernardoCheptou, Pierre-OlivierÖrdög, AttilaCherif, EmiraOrfanoudakis, MichailChertov, OlegOrlova-Bienkowskaja, Marina J.Chialva, MatteoOrtega, JohnChiapella, JorgeOrton, Thomas J.Chiarini, LuigiOrtúñez, EmmaChien, Mei-FangOsborne, Bruce A.Chiesi, MartaOslovsky, VladimirChiluwal, AnujOstaszewska-Bugajska, MonikaChim, Bee KhimOstręga, AnnaChin, Joong HyounOstrowska, AgnieszkaChin, Young-WonOszako, TomaszChini, AndreaOtagaki, ShungoChiou, Chun-TangOttaiano, LuciaChirumbolo, SalvatoreOtto, Daniel P.Chitambar, John J.Otulak-Kozieł, KatarzynaChitarra, WalterÖtvös, KrisztinaChiumenti, MichelaOudemans, PeterChiurazzi, MaurizioOugham, HelenChiwa, MasaakiOuyabe, MichelChmara, RafałOuzounidou, GeorgiaChmielowska-Bąk, JagnaOwens, Daniel K.Chmura, DamianOzarowski, MarcinCho, Won KyongPaci, MaurizioCho, Yong-GuPaciolla, CostantinoChobot, VladimírPaço, AnaChoi, DongsuPăcurar, FlorinChoi, Hyo GilPacuta, VladimirChoi, Won-GyuPaczos-Grzeda, EdytaChojnacka-Ożga, LonginaPadoan, ElioChorianopoulou, Styliani N.Pagán, IsraelChoromanska, AnnaPagano, MarioChorostecki, UcielPaganová, VieraChoudhury, Swarup RoyPagliarani, ChiaraChowdhury, KamalPagnotta, Mario A.Christensen, AlanPahwa, RomaChristoffers, MichaelPais, Maria SaloméChristopoulou, AnastasiaPaixao Coelho, Jose AugustoChrysargyris, AntoniosPająk, PaulinaChrzanowski, ŁukaszPajoro, AliceChung, HsiaohangPál, MagdaChung, KwiMiPalacios, José-ManuelChwil, MirosławaPalla, FrancoChyau, Charng-CherngPalma, DanielaCiąćka, KatarzynaPalmer, AndrewCianfaglione, KevinPalmero-Llamas, DanielCiarmiello, LoredanaPalmgren, Michael GCicero, ArrigoPaltinean, RamonaCid, ClémentPampana, SilviaCiereszko, IwonaPan, Bruce S.Cilas, ChristianPan, Yong-BaoCillo, FabrizioPanayotis, TrigasCimpoiu, ClaudiaPanayotova, VeselinaCioć, MonikaPanday, DineshCipakova, IngridPandey, Avinash ChandraCires, EduardoPandey, Dhananjay K.Cizkova, JanaPaneque, MarinaClark, Christopher A.Pang, NaClark, Natalie MPangallo, DomenicoClaudia, Salanță LianaPankievicz, Vânia C. S.Clavijo-McCormick, AndreaPantelides, IakovosCleavitt, Natalie L.Panteris, EmmanuelClemente, RafaelPapacek, StepanClericuzio, MarcoPapafotiou, MariaCliment, JoséPapageorgiou, AristotelisClotault, JérémyPapathanasiou, FokionCocucci, MaurizioPapathanasiou, VasillisCogoni, AnnalenaPapenbrock, JuttaCogoni, DonatellaPapini, AlessioCohen, YigalPaplomatas, E. J.Coimbra, SilviaPaponov, Ivan A.Coldea, Teodora EmiliaPappa, AglaiaCole, Logan W.Paradjikovic, NadaColicchio, JackParajuli, SarojColmenero-Flores, José M.Paredes-López, OctavioCombier, Jean-philippeParent, Léon-ÉtienneCominelli, EleonoraParis, RobertaCommisso, MauroPark, Chong-WookCondon, TonyPark, Il-KwonConesa, María R.Park, InkyuCongestri, RobertaPark, Kyung-MokConklin, Patricia L.Park, Myoung RyoulConn, Caitlin E.Park, Sang WookConsonni, GabriellaPark, Woong JuneConstantinescu-Aruxandei, DianaParkar, Shanthi G.Constantinidis, TheophanisParker, Steven L.Conti, HeatherParrotta, LuigiCook, StephenParsaeimehr, AliCopeland, CharlesParsons, JasonCopes, Josh T.Parus, AnnaCopetta, AndreaParveen, IffatCopic, AlenkaPârvu, Alina ElenaCopolovici, LucianPârvu, MarcelCordeiro, NereidaPascual-Seva, NúriaCordoba-Diaz, D.Pasin, FabioCordovilla, María del PilarPassera, AlessandroCorio-Costet, Marie-FrancePastenes, ClaudioCornejo, PabloPasternak, TarasCorrado, GiandomenicoPatel, Jai SinghCorredoira, ElenaPaterson, AndrewCorreia, Carlos M.Pathak, AshishCorreia, PedroPathak, BhuvanCorreia, SofiaPathak, Tapan B.Corsetto, PaolaPatterson, EricCortes, Andres J.Păucean, AdrianaCosmin Mihai, VesaPaudel, IndiraCosoveanu, AndreeaPaulen, OlegCosta De Camargo, AdrianoPaulette, LauraCosta, David SchellenbergerPavela, RomanCosta, JoanaPavic, ValentinaCostantino, PaoloPavlov, AtanasCostanza, BaldisserottoPavšič, MihaCota-Ruiz, KeniPaweł’kowicz, Magdalena ECourdavault, VincentPawlak, AleksandraCoutinho, Henrique Douglas MeloPawlik-Skowrońka, BarbaraCouturier, JeremyPawłowicz, IzabelaCouzinet-Mossion, AureliePawlowski, Michelle L.Covarelli, LorenzoPayton-Stewart, FlorastinaCovo, ShayPayumo, JaneCox, Cymon J.Pazos-Navarro, MariaCox, RobertPecio, AlicjaCozzolino, EugenioPedersen, HenrikCreamer, RebeccaPeer, Wendy AnnCrespi, Martín D.Peerzada, Arslan MasoodCrevillén, PedroPeixe, AugustoCrisp, PeterPellegrini, MarikaCristaldi, AntonioPeltier, GillesCristaudo, Antonia EgidiaPeña-Gómez, NatalyCristiano, GiuseppePeng, CongyueCrivellaro, AlanPeng, FoenCrupi, PasqualePeng, GaryCsaba, MáthéPeng, XianjunCséplő, ÁgnesPennerman, Kayla K.Csorba, TiborPentecost, AllanCuadrado, AngelesPerdomo, Juan AlejandroCubillos, FranciscoPereira, CarlaCuellar, SaraPereira, CláudiaCuesta, CandelaPereira, JoseCuevas, JuliánPereira, Olívia R.Cui, RongfengPereira, Susana S.Cullis, ChristopherPereira, TiagoCumming, JonathanPérez De Castro, AnaCupellini, LorenzoPerez De Souza, LeonardoCurtis, PeterPerez, FelipeCvrčková, FatimaPérez-Álvarez, Jose AngelCwalina-Ambroziak, BożenaPérez-Fernández, MaríaCzajkowski, RobertPérez-García, Francisco J.Czekała, WojciechPérez-López, EdelCzembor, Jerzy HenrykPérez-Marcos, MaríaCzerwinska, MonikaPérez-Vargas, IsraelD’Addabbo, TrifonePerlikowski, DawidD’Alessandro, StefanoPerlin, MichaelD’Auria, MaurizioPernisová, MarkétaD’Urso, GildaPernot, ClémentineDa Silva, Joyce Kelly R.Perochon, AlexandreDa Silva, Washington LuisPerotto, Humberto L.Daba, Ketema A.Perreau, FrancoisDabrowski, PiotrPerrella, GiorgioDag, ArnonPerrine-Walker, FrancineDagnon, SoleyaPerrino, EnricoDahlin, PaulPerronne, RémiDai, YuminPerry, SharynDalCorso, GiovanniPers-Kamczyc, EmiliaDall’Acqua, StefanoPestsova, Elena G.Dall’Osto, LucaPetanidou, TheodoraDalziell, EmmaPetek, MarkoDamalas, Christos A.Peter, KatherineDamodaran, SureshPeters, KristianDaniel, HervéPetrakis, Panos V.Danova, KalinaPetrášek, JanDaras, GerasimosPetriccione, MilenaDarbani, BehroozPetricevich, VeraDarch, TeganPetropoulos, SpyridonDarenskaya, M. A.Petrova, MariaDarie-Nita, Raluca NicoletaPetrová, ŠárkaDarkó, ÉvaPetrova, SlaveyaDarnell, RossPetrussa, ElisaDaroch, MaurycyPetrzik, KarelDarras, AnastasiosPetti, CarloalbertoDasgupta, KasturiPfeilmeier, SebastianDastogeer, KhondokerPfister, BarbaraDatta, RahulPhillips, TimDaugrois, Jean-HeinrichPhukan, Ujjal JyotiDaunay, Marie-ChristinePiasecki, CristianoDauwe, RebeccaPiątczak, EwelinaDavid, LuminitaPicardi, ErnestoDavid, Teresa S.Piccinelli, Anna LisaDavydenko, KaterynaPiccolella, SimonaDay, DavidPicq, SandrineDayan, FranckPiedras, PedroDaygon, DaraPiepenbring, MeikeDe Andrade Moral, RafaelPiernik, AgnieszkaDe Bellis, LuigiPieroni, AndreaDe Carlo, AnnaPieters, Alejandro J.De Caroli, MonicaPietrantonio, Patricia VDe Carvalho, Ricardo CruzPietrowska-Borek, MałgorzataDe Feo, VincenzoPietrzak, WioletaDe Freitas Fraga, Hugo PachecoPignone, DomenicoDe Gara, LauraPilarek, MaciejDe Haro, Luis AlejandroPilkington, LisaDe Heredia, Unai LópezPilon-Smits, Elizabeth A. H.De Jesus Pereira Godinho Velada, IsabelPilu, RobertoDe La Torre, AmandaPiluzza, GiannellaDe Luca, FrancescaPiñero-Dalmau, DanielDe Lurdes Inácio, MariaPinker, InaDe Marco, AnnaPinnola, AlbertaDe Masi, LuigiPintea, AdelaDe Mol, FriederikePinto, ErnaniDe Oliveira, Mara Elisa SoaresPinto, LorisDe Palma, LauraPinzaru, IuliaDe Paolis, AngeloPipan, BarbaraDe Rosa, Maria CristinaPiro, GabriellaDe Sousa, Damião PergentinoPirona, RaulDe Tullio, Mario C.Pirvu, LuciaDe Vienne, DominiquePisciotta, AntoninoDe Vita, DanielaPiscopo, MarinaDe Vries, JanPistelli, LauraDebnath, SamirPittman, Jon K.Deconinck, KoenPivić, RadmilaDegani , Ofir Płachno, BartoszDegola, FrancescaPlader, WojciechDehelean, CristinaPlastina, PierluigiDehghan, EsmaeilPŁażek, AgnieszkaDeineko, Elena VictorovnaPliego-Alfaro, FernandoDel Castillo-Santaella, TeresaPlíhal, OndřejDel Genio, CharoPlíhalová, LucieDel Pozo, J. CarlosPlitta-Michalak, BeataDel Río Celestino, MercedesPlouviez, MaxenceDélano-Frier, John PaulPniewski, TomaszDelgado Santander, RicardoPoblete, TomasDelhomme, NicolasPoblete-Grant, PatriciaDelis, CostasPocock, TessaDello Ioio, RaffaelePodar, DorinaDel-Rio Celestino, MercedesPodgórska, AnnaDeltsidis, AngelosPodwyszynska, MalgorzataDemasi, SoniaPohl, PawelDen Berg, Cássio VanPoinar, GeorgeDeng, ZhanaoPoirot, MarcDenisow, BozenaPoljak, IgorDepa, ŁukaszPoljuha, DanijelaDepeint, FlorePolkowska-Kowalczyk, LidiaDeshmukh, RupeshPollastrini, MartinaDespland, EmmaPollastro, FedericaDevecchi, MarcoPolo, JavierDever, JanePommerrenig, BenjaminDewdney, Megan M.Pongrac, PaulaDeWitt, Thomas J.Ponnu, JathishDhakal, DipeshPonte, NunoDhandapani, VigneshPoojari, SudarsanaDhankher, Om ParkashPoór, PéterDhiman, SaurabhPopescu, IulianaDhungana, SanjeevPopłońska, KatarzynaDi Bene, ClaudiaPopova, AntoanetaDi Giacomo, SilviaPopova, Elena V.Di Mambro, RiccardoPopova, Milena P.Di Martino, CatelloPorceddu, MarcoDi Matteo, AdelePortillo-Estrada, MiguelDi Mola, IdaPoschenrieder, CharlotteDi Salle, AnnaPoslušná, JanaDi Sanzo, RosaPospisil, JiriDi Serio, FrancescoPospisil, TomasDi Venere, DonatoPotter, DanielDíaz Esteban, MarioPottosin, IgorDíaz-Espejo, AntonioPoulicek, Mathieu E.A.Diaz-Garcia, LuisPowell, Ann L. T.Dickey, AaronPower, Robert CDictor, Marie-ChristinePozzi, CarloDiederichsen, AxelPrabakaran, MayakrishnanDifonzo, GrazianaPradeep Kumar, SudheeranDijkwel, PaulPrado-Cabrero, AlfonsoDilfuza, EgamberdievaPrasad, AnkushDimandja, Jean-MariePrasad, KasavajhalaDimario, Robert J.Pratley, JimDimcheva, VanyaPrats, María SoledadDimkpa, ChristianPrerostova, Mgr. SylvaDING, CHENPrieto Garcia, JoseDing, JingyiPrieto, MiguelDing, LeiPrince, Silvas JDinica, Rodica-MihaelaPritchard, HughDinkins, RandyPrkić, AnteDireito, RosaPROBST, AlineDitta, AllahProestos, CharalamposDivashuk, MikhailProm, LouisDjidonou, DesirePrudent, MarionDjilianov, DimitarPrüfer, DirkDługosz-Grochowska, OlgaPrusky, DovDo Paço, AnaPrzybył, Jarosław L.Do, Yi-YinPrzybysz, ArkadiuszDoblin, MonikaPszczółkowska, AgnieszkaDobránszki, JuditPuchalka, RadoslawDobrikova, AneliaPucker, BoasDobrowolska, GrażynaPudziuvelyte, LaurynaDocimo, TeresaPuglia, GiuseppeDoddapattar, PrakashPuglielli, GiacomoDolashka, PavlinaPuglisi, IvanaDolatabadian, AriaPugovics, OsvaldsDoley, DavidPusztahelyi, TündeDoležal, KarelPycia, KarolinaDolgikh, ElenaQaderi, MirwaisDolors Serret, Maria MolinsQi, AimingDolzhenko, Anton V.Qiang, ShengDombrovsky, AvivQiao, PengfeiDomingo, ConchaQin, HongminDomingues, Douglas SilvaQin, WenshengDominguez, EloisaQing, Lin-senDominguez, EvaQiu, YongjianDomisch, TimoQualset, Calvin O.Domozych, David S.Quatrini, PaolaDonaire, LiviaQuéro, AnthonyDong, FaqinQuesada Pérez, VíctorDong, HongxuQuesada, Miguel A.Dong, JieQuintanilla, MarisolDong, L. (Lemeng)Quiroga, GabrielaDonno, DarioRabara, RoelDonze-Reiner, TeresaRabot, AmélieDordas, Christos ARaccuia, Salvatore AntoninoDoria, EnricoRace, MarcoDos Santos, Maria Clara VieiraRaddi, SabrinaDOSOKY, NOURARadini, AnitaDouda, OndřejRadosavljevic, IvanDownie, J. AllanRadotic, KsenijaDoxa, AggelikiRadu, FierascuDoyle, SiamsaRadulov, IsidoraDrava, GiulianaRafińska, KatarzynaDresler, SławomirRagazinskiene, OnaDriouich, AzeddineRago, DanielaDrucker, MartinRagonezi, CarlaDu, HaishunRaguso, RobertDu, KeRahemi, AlirezaDu, MinminRahikainen, MoonaDu, XinRahman, Md. AtikurDu, ZhiyanRai, VineetaDuan, JialeiRaikhy, GauravDuan, Yong P.RAILLARD, CécileDuarte, BernardoRajarapu, Swapna PriyaDuarte, Gustavo TurquetoRajesh, YarraDuarte, Maria CristinaRajniak, JakubDubois, LenaRaju, RiteshDubreucq, BertrandRakotondrafara, AurelieDubrovina, AlexandraRamachandran, SrinivasanDuckett, Jeffrey G.Raman, HarshDucsay, LadislavRamana Pidatala, VenkataDuda, Marcel MateiRamasamy, ManiDufossé, LaurentRamegowda, VenkategowdaDugé De Bernonville, ThomasRamírez-Parra, ElenaDuijn, Bert VanRamos, Patricia A.B.Duke, Stephen O.Ramos, PawełDulloo, Mohammad EhsanRamos, SebastianDumenyo, KorsiRandall, Stephen K.Dumičić, GvozdenRanucci, DavidDumitru, GabrielaRapeanu, GabrielaDumont, JosephRaposo, RosaDunemann, FrankRaseduzzaman, MdDunić, Jasenka AntunovićRashotte, Aaron M.Dunn, Michael F.Rasiukevičiūtė, NeringaDupuis, JulianRasmussen, SorenDurand, PaulineRaspé, OlivierDurgo, KsenijaRastija, VesnaDuruflé, HaroldRastogi, AnshuDusenge, Mirindi EricRatajczak, KarolinaDushenkov, VyacheslavRatajewski, MarcinDutu, LigiaRatcliffe, SophiaDuvnjak, TomislavRato, Ana ElisaDwiyanti, Maria StefanieRattenholl, AnkeDyderski, Marcin K.Raúl, Avila-SosaDziadas, Mariusz TomaszRavelonandro, MichelDzida, KatarzynaRavin, Nikolai V.Dziurka, MichalRawat, SwatiE. Harms, NathanRaynor, EdwardEamens, Andrew L.Rayon, CatherineEardly, BertrandRažná, KatarínaEaves, JamesRead, David AlanEbadollahi, AsgarRead, John J.Ebert, Andreas W.Rebollo-Hernanz, MiguelEbert, Timothy AReczyńska, KamilaEbihara, AtsushiReda, Abou-ShanabEderli, LuisaReda, MałgorzataEfthimiadou, AspasiaRedden, RobertEggler, AimeeReddy, UmeshEichberg, CarstenReed, Kevin F.M.Eilers, Elisabeth JohannaReglinski, TonyEisenback, Jonathan D.Regni, LucaEl-Aarag, Bishoy Y.A.Rehrig, ErinElansary, Hosam O.Reig, GemmaElbasyoni, IbrahimReinhart, Kurt O.Elbeaino, TouficReis, CarlosEl-Esawi, Mohamed A.Reisdorf-Cren, MichèleElfalleh, WalidRemke, MichaelEliášová, KateřinaRemm, LiinaElibox, WinstonRenault, SylvieEliopoulos, Panagiotis A.Renčo, MarekElkins, KellyRengasamy, KannanEllis, RichardRengefors, KarinEl-Ramady, HassanRengo, SandroEl-Seedi, HeshamRenner, Matt A.M.Elshafie, Hazem S.Resende, Diana I.S.P.Elshamy, AbdelsamedResentini, FrancescaEl-Sharkawy, IslamRestivo, Francesco M.Endre, GabriellaRestuccia, AlessiaEndresen, DagReuveni, MosheEndress, PeterRevilla, EugenioEngelberth, JurgenRewicz, AgnieszkaEngelmann, FlorentRey, LuisEparvier, VéroniqueRey, María-DoloresEras, JordiRey, PascalErickson, David L.Reyes-Chilpa, RicardoErinle, KehindeRezania, ShahabaldinEsatbeyolgu, TubaRhazi, LarbiEshel, AmramRho, HyungminEsmaeel, QassimRial, CarlosEspen, LucaRibaudo, GiovanniEspenberg, MikkRibeiro, HelenaEsposito, SergioRibeiro-Barros, Ana I.Esquivel, BaldomeroRicelli, AlessandraEsser, Kjell B.Richards, Christopher M.Esteve-Romero, JosepRiddick, Eric WellingtonEsumi, TomoyaRienth, MarkusEszter, Hubainé TóthRigano, Maria ManuelaEudes, AymerickRigas, StamatisÉva, CsabaRigó, GáborEvangelisti, EdouardRikkerink, ErikEves-van Den Akker, SebastianRinaldi, Andrea C.Ezell, Andrew W.Rios, Juan JoseEzzati, SattarRissel, DagmarFabiani, RobertoRiva, AntonellaFacchiano, AngeloRival, AlainFadón, EricaRizzo, RobertoFagerstedt, KurtRoach, ThomasFail, JózsefRobakowski, PiotrFailla, SabinaRoberto, Sergio RuffoFajkus, JiříRobertovna, Garsiya EkaterinaFalcão, Soraia I.Roberts, Wade RFalistocco, EgiziaRobinson, ToddFall, MamadouRobles, Eva SánchezFallacara, Dawn M.Robles, PedroFan, LiuminRoccuzzo, GiancarloFan, LushengRodamilans, BernardoFanelli, ValentinaRodrigo-Comino, JesusFanfarillo, E.Rodrigues, FranciscaFang, DavidRodrigues-Pousada, Renato AlbertoFang, FrancisRodríguez González, ÁlvaroFang, Wei-TaRodríguez Villanueva, JavierFanourakis, DimitriosRodríguez, Víctor ManuelFantinato, EdyRodríguez-Flores, M ShantalFaralli, MicheleRodríguez-Jimenes, GuadalupeFaraone, NicolettaRodriguez-Rajo, Francisco JavierFardet, AnthonyRodríguez-Rosales, M. PilarFaria, Jorge M.S.Rodríguez-Yoldi, María JesúsFarina, VittorioRogozhin, EugeneFarinos, H. MerleRohila, JaiFarkas, ÁgnesRohn, SaschaFarman, MarkRojas, ClemenciaFarneti, BrianRojas, RomeoFarzaneh, VahidRolbiecki, LeszekFascella, GiancarloRolo, VictorFavas, PauloRomaneckas, KestutisFavre, AdrienRomano, DanielaFavre-Réguillon, AlainRomanowska-Duda, ZdzisławaFazekašová, DanicaRomeiras, Maria M.Fazio, AlessiaRomero, IreneFedderwitz, FraukeRomina Alina, Marc (Vlaic)Feder, FrédéricRomuli, SebastianFedriani, Jose M.Rondanini, Deborah P.Fehér, AttilaRonga, DomenicoFeledyn-Szewczyk, BeataRoni, Md Zohurul KadirFenselau, CatherineRonzan, MarilenaFenu, GiuseppeRood, Stewart B.Feraru, ElenaRopelewska, EwaFereres, AlbertoRosa, CristinaFerguson, JohnRosa, TundisFernandes, Elizabeth S.Roscoe, ThomasFernandez, EduardoRosell, Rosemarie C.Fernandez, JessieRosen, CarlFernández, Juan A.Ross, Ian LFernández, María DoloresRoss, KellyFernandez, PaulaRossi, LorenzoFernández-González, FedericoRostoks, NilsFernández-Martínez, MarcosRothballer, MichaelFerrante, AntonioRotolo, CaterinaFerrante, PatriziaRoubelakis-Angelakis, Kalliopi A.Ferrario-Méry, SylvieRoulia, MariaFerrater, JedelizaRouphael, YoussefFerreira, Juan JoséRoussos, Peter A.Ferreira, MarceloRoutray, PratyushFerrero-Serrano, ÁngelRovere, Federica DellaFerrio Díaz, Juan PedroRowland, Lisa JFetcher, NedRoy, MélanieFett-Neto, Arthur GermanoRoy, NehaFialová, SilviaRoychowdhury, RajibFidan, HafizeRoyo-Esnal, AritzFidelibus, MatthewRóżalska, SylwiaField, RichardRozentsvet, Olga A.Fierascu, IrinaRozhon, WilfriedFIÉVET, Julie B.Różyło, KrzysztofFigaj, DonataRu, SushanFigueiredo, AndreiaRubinowska, KatarzynaFigueiredo, DuarteRubio, LourdesFilek, MariaRucińska-Sobkowiak, RenataFilion, MartinRudnóy, SzabolcsFilip, AdrianaRuelland, EricFilipecki, MarcinRufo, LourdesFiliz, ErtugrulRugini, EddoFilkov, VladimirRuiz Téllez, TrinidadFinetti-Sialer, Mariella MatildeRuiz, LeticiaFinlayson, ScottRuiz-Cruz, SaulFiore, Maria CarolaRuiz-Garcia, LeticiaFirlak, MelikeRumbou, ArtemisFischer, LukášRumyantseva, NatalyaFischer-Fodor, EvaRungis, DainisFischer-Schrader, KatrinRunion, George BrettFitsiou, EleniRurek, MichalFladung, MatthiasRussi, LuigiFlamini, GuidoRusso, AlessandraFlenniken, Michelle L.Russo, DanielaFletcher, KyleRusso, Maria ElenaFlinn, BarryRustioni, LauraFlores-Félix, J.D.Rusu, TeodorFodor, FerencRutten, TwanFoereid, BenteRyabova, LyubaFogacci, FedericaRybczyńska-Tkaczyk, KamilaFois, MauroRybka, KrystynaFojtová, MiloslavaRydgren, KnutFonseca, JoseRymen, BartFontés, MichelRyszka, PrzemysławForján, RubénRyu, JaihyunkFormentin, ElideRyu, Kwon-YulForni, CinziaRyu, Stephen BeungtaeForoud, Nora A.S. Tørresen, KirstenForreiter, ChristophSabater, BartolomeForrer, Hans-RudolfSabatino, LeoForti, LucaSabbadini, SilviaFouracre, JimSabella, ErikaFrabboni, LauraSabovljevic, MarkoFracchiolla, MarianoSadgrove, NicholasFragkostefanakis, SotiriosSadhukhan, AyanFraguas-Sánchez, Ana IsabelSadka, AviFrances, SussmilchSadot, EinatFrancesca, SilvanaSaha, RajibFranchi, ElisabettaSahu, PranavFrancis, FredericSahuquillo, Elvira BalbuenaFranco, Albina R.Saiardi, AdolfoFranić, MarioSaito, KatsuharuFranke, KatrinSajeva, MaurizioFranken, PhillipSakai, AtsushiFranklin, JenniferSakamoto, AyakoFranz, ChlodwigSakamoto, MasahiroFrąszczak, BarbaraSakamoto, MasaruFray, RupertSakar, GermánFredotović, ŽeljanaSako, KaoriFreire, João A.Sala, FlorinFreire, M. SoniaSala, TeaFrels, KatherineSalachna, PiotrFrey, MaximilianSalaheen, SerajusFriedt, WolfgangSalamon, IvanFrolov, AndrejSalamon-Albert, ÉvaFrolova, Tatiana S.Šalamún, PeterFrugis, GiovannaSalánki, KatalinFruk, GoranSalanţă, Liana ClaudiaFry, William EarlSalas, José BlancoFu, FuyouSalas, Maricarmen C.Fu, JianmingSalazar, Juan RodrigoFu, PengSaleh-E-In, Md. MoshfekusFu, XinyuSalehin, MohammadFu, Yong-BiSalimonti, AmeliaFuchs, MarcSalomaki, Eric D.Fuentes, SigfredoSalome, PatriceFujii, ShoSalopek-Sondi, BrankaFujii, YoshiharuŠamec, DunjaFujimoto, RyoSamigullin, Tahir H.Fujino, TakeshiSamira, RozalynneFujita, MasayukiSamson, GuyFujiwara, ShokoSan Bautista, AlbertoFukuda, YukiSánchez Del Pino, Manuel M.Fukudome, AkihitoSanchez Muñoz, RaulFukunaga, KenjiSánchez Torres, PalomaFukushima, AtsushiSanchez Vallet, AndreaFunada, RyoSánchez, Diego HernánFunnekotter, BrynSánchez-García, MaríaFyhrquist, PiaSanchez-Guerrero, Maria CruzGabbia, DanielaSánchez-Mata, DanielGaberščik, AlenkaSanchez-Moreiras, AdelaGabr, MoustafaSanchez-Rodriguez, AntonioGabriela Goia, IrinaSánchez-Romero, CarolinaGabriele, MorenaSandalio, Luisa MaríaGabryś, BeataSandoval, MaricarmenGadaleta, AgataSangiovanni, EnricoGage, KarlaSangireddy, Sasikiran ReddyGaj, MalgorzataSanjeewa, Kalu Kapuge AsankaGaj, RenataSano, NaotoGajc-Wolska, JaninaSansberro, Pedro AGajdošová, AlenaSantabarbara, StefanoGalán De Mera, AntonioSantaeufemia, SergioGalanakis, Charis M.Santamaria, PietroGalbiati, MassimoSantana, MargaridaGalbraith, DavidSantangelo, EnricoGalea, VictorSantiago-Rodriguez, Tasha MarieGalek, RenataSantini, AntonelloGaliba, GaborSantos López, Jorge ArturoGalic, AnteSantos, CarlaGallaher, Sean DavidSantos, MilaGalland, PaulSanz-Alférez, SoledadGallé, Ágnes AnnaSanzani, Simona MariannaGallego, Juan Carlos AlíasSara, MontanariGallego-Fernández, Juan B.Saracchi, MarcoGallego-Tévar, BlancaSarasan, ViswambharanGalstyan, AnahitSârbu, CostelGamarra, RobertoŠárka, EvženGambino, GiorgioSarrou, EiriniGamboa-deBuen, AliciaSasanelli, NicolaGan, YinboSas-Paszt, LidiaGanesan, PalanivelSassenhagen, IngridGanguly, AnindyaSatake, MasayukiGao, BeiSatish, LakkakulaGao, GarySato, ShinyaGao, RuiminSato, YasushiGaragounis, ConstantineSatoh, ShigeruGarbe, Leif- AlexanderSator-Katzenschlager, SabineGarcía Martín, Juan FranciscoSattar, SampurnaGarcía, Mary Paz GonzálezSattler, ScottGarcia, SoniaSaurav, KumarGarcía-González, IgnacioSauvage, ChristopherGarcía-González, Marta EvaSavazzini, FedericaGarcia-Molina, AntoniSavchenko, Tatyana V.García-Pérez, PascualSavenkov, EugeneGarcia-Ruiz, HernanSavić, JelenaGardiner, JasonSavini, SaraGardiner, Laura-JayneSavoi, StefaniaGardlik, RomanSavvidou, MariaGardner, AndrewSawicka, BarbaraGarita-Cambronero, JersonSawicki, JakubGarmendia, IdoiaSawicki, TomaszGarnatje, TeresaSazonova, Margarita A.Garrido, AdriánScala, ValeriaGarvin, DavidScalabrin, ElisaGarzoli, StefaniaScalabrin, SimoneGascó, AntonioScarpa, Grazia MariaGašić, UrošScartazza, AndreaGašo-Sokač, DajanaScepanovic, MajaGasparatos, DionisiosSchachtman, DanielGasparis, SebastianSchäfer, MartinGates, ColinSchaller, JörgGatz, ChristianeSchandry, NiklasGavilán, Rosario G.Scharff, LarsGavrila, Adina IonutaSchena, LeonardoGawlik-Dziki, UrszulaSchenck, Craig A.Gaworski, MarekScherer, GüntherGawroński, PiotrScheuring, DavidGebauer, RomanSchiavon, MichelaGebre, GirmaSchiermeyer, AndreasGecheva, GanaSchilling, SusanneGehring, ChristophSchinkovitz, AndreasGeisler, MattSchinnerl, JohannGendaszewska-Darmach, EdytaSchmidt, DominikGenga, AnnamariaSchmidt, LilianGenovese, CarloSchmidt, TravisGenovese, ClaudiaSchmitt-Keichinger, CorinneGent, MartinSchneider, AnjaGentile, AlessandraSchneider, RenéGentili, RodolfoSchoenaers, SébastjenGeorgia, NtatsiScholz, MiklasGeorgiev, VasilSchrader, AndreaGeorgiev, YordanSchrey, AaronGeorgieva, OlgaSchripsema, JanGerbeau-Pissot, PatriciaSchröder, PeterGerke, JörgSchroeder, HilkeGerm, MatejaSchroeder, NathanGermano, Maria PaolaSchulz, AlexanderGesch, RussSchulz, MargotGesell, AndreasSchumann, UlrikeGhadieh, Hilda E.Schuster, GadiGhatak, ArindamSchwab, RebeccaGhazanfar, ShahinaSchwachtje, JensGhignone, StefanoSchwan, WilliamGhilardi Lago, João HenriqueSchwarz, ErikaGhimire, BalkrishnaSchwarzländer, MarkusGhirardo, AndreaScotti, NunziaGhiulai, RoxanaScotton, MicheleGiacometti, AndreaScrano, LauraGianinetti, AlbertoSebastiani, FedericoGiannakou, IoannisSeben, VladimirGiannakoula, AnastasiaSeca, Ana Maria Loureiro DaGiannoutsou, EleniSegami, ShojiGiarola, ValentinoSeidel, ThorstenGil, MinchanSeidl-Adams, IrmgardGill, UpinderSekara, AgnieszkaGillman, JasonSekine, MasamiGil-Pelegrín, EustaquioSeleiman, Mahmoud F.Gilroy, SimonSeligmann, HervéGimenez, EstelaSelvaraj, Michael GomezGiordani, PaoloSena, KentonGiorni, PaolaSengupta, BidishaGiovannetti, MarcoSengupta-Gopalan, ChampaGiovannini, AnnalisaŞenilă, MarinGiraldo, Martha CeciliaSeo, Hak SooGiroux, MikeSeo, Young-SuGitzendanner, MattSera, BozenaGiussani, BarbaraSerafini Fracassini, DonatellaGladish, Daniel K.Seralini, Gilles-EricGlanc, MatoušSerba, DesalegnGlassop, DonnaSergeant, KjellGlazińska, PaulinaSergio, LucreziaGłowacka, AleksandraSerio, FrancescoGniazdowska, AgnieszkaSerrano, IreneGoc, AnnaSestili, FrancescoGoddard, Mary-LorèneSestili, SaraGoldenkova-Pavlova, IrinaSestras, RaduGolding, John B.Setyaningsih, WidiastutiGoldschmidt, Eliezer E.Setzer, William N.Gollin, DouglasSeymour, NikkiGolob, AleksandraSgherri, CristinaGolubkina, NadezhdaSgorbini, BarbaraGomes, SóniaShabala, LanaGómez López, PedroShafiq, SarfrazGómez, Gustavo G.Shahba, Mohamed A.Gómez-Mena, ConcepciónShahin, ArwaGonçalves, José Francisco De C.Shahinnia, FahimehGonda, SandorShaikhali, JehadGonzález García, VicenteShapiguzov, AlexeyGonzález, Ana M.Sharbrough, JoelGonzalez-Guzman, MiguelSharma, AnketGonzález-Ibeas, DanielSharma, KavitaGonzález-Laredo, Rubén FranciscoSharma, NiharikaGoodwin, SteveSharpe, ShaunGoraj, WeronikaSharwood, RobertGorchov, DavidShchennikova, AnnaGorgan, LucianShcherban, Andrey B.Gori, AntonellaShekasteband, RezaGorini, FrancescaShen, JianqiangGorji, Sara GhorbaniSheng, RuilongGornas, PawelShepherd, Lara DGorpenchenko, Tatiana Y.Sherstneva, OksanaGospodarek, JaninaShi, GongjunGossman, ToniShibaeva, Tatjana G.Goto, EijiShiina, TakashiGotor, Alicia AyerdiShilev, StefanGott, Jonatha M.Shim, DonghwanGötz, Klaus-PeterShimelis, HusseinGousset-Dupont, AurélieShimojima, MieGouveia, Alexandra M.Shimomura, HirofumiGovindjee, GovindjeeShinano, TakuroGoyal, EtikaShinde, SuhasGoyali, Juran C.Shinozaki, JunichiGozdowski, DariuszShinozaki, YoshihitoGrabarczyk, MałgorzataSHIRO, SokichiGrabau, Larry J.Shiryaev, Anton G.Grabau, ZaneShishova, Maria F.Grabelnych, Olga I.Shoeva, OlesyaGraham, JulieShtein, IlanaGramss, GerhardShuh Ichi, NishikawaGrando, StellaShukla, MukundGranica, SebastianShukla, Pushp SheelGrassi, FabrizioSiatka, TomasGreenberg, AnthonySiatkowski, IdziGregor, ThomasŠibík, JozefGregorio, Gálvez-ValdiviesoSibin, YuGresta, FrancescoŠic Žlabur, JanaGriesser, MichaelaSicari, VincenzoGriffith, M. PatrickSieber, Alisa NaomiGrigore, Marius N.Siemianowski, OskarGristina, Alessandro SilvestreSierro, NicolasGródek-Szostak, ZofiaSikorska, AnnaGrones, PeterSilaghi-Dumitrescu, RaduGrossi, GiancarloŠiler, BranislavGroten, KarinŠilha, DavidGruhlke, Martin C. H.Silva Dias, LuísGrzebelus, DariuszSilva, GoncaloGrzebelus, EwaSilva, Maria HelenaGrzegorczyk, IzabelaSilva, Roberto NascimentoGrzegorczyk-Karolak, IzabelaSilvestri, CristianGrzesiak, MaciejSima, Nicusor-FlaviusGrzywacz, AgataSima, Rodica MariaGu, LipingSimeonov, VasilGu, XingyouSimirgiotis, MarioGuadagno, CarmelaSimkin, AndrewGuarino, CarmineSimm, StefanGuarino, FrancescoSimões, FernandaGuarino, SalvatoreSimões, Marta FilipaGuerra, MiguelSimonini, SaraGuerra-Sanchez, GuadalupeSimonsen, Henrik ToftGuevara González, RamónSimova-Stoilova, LyudmilaGugała, MarekSimpson, CraigGuido, LuisSimpson, Richard J.Guidolotti, GabrieleSims, JamesGuillotin, BrunoŠindlerová, LenkaGuldin, James M.Singer, Stacy D.Gullì, MariolinaSingh, GurbirGunjača, JerkoSingh, ManjulGuo, Ming-quanSingh, Rupesh KumarGuo, YunhuiSingh, UpendraGuo, ZhanjunSingh-Cundy, AnuGupta, CharuSiniscalchi, Carolina M.Gururani, Mayank AnandSinjushin, Andrey A.Gurusamy, RamanSitarek, PrzemysławGustafson, Danny J.Sitko, KrzysztofGustin, JefferySivanesan, IyyakkannuGuttieri, Mary J.Sivarajan, Sajeevan RadhaGuy, Charles L.Skała, EwaGuyot, RomainSkalicky, MilanGuzmán, José MiguelSkalska-Kamińska, AgnieszkaGwathmey, OwenSkelton, RobertGyöngyvér, MaraSkenderidis, ProdromosGyula, PéterSkendi, AdrianaHa, Bo-KeunSkouras, Panagiotis J.Habili, NuredinSkowrońska, MonikaHack, EthanSkre, OddvarHadidid, AhmedSkrt, MihaelaHafez, SaadSkrypnik, LiubovHage-Ahmed, KarinSkrzypek, EdytaHagidimitriou, MariannaSkuhrovec, JiříHaider, SaqlainSławski, MarekHaig, DavidŚlesak, HalinaHaigh, AnthonySlimestad, RuneHaile, JemaneshŚlipiko, MonikaHakkenberg, ChristopherSliwinska, ElwiraHalitschke, RaykoŚliwińska-Wilczewska, SylwiaHallmann, EwelinaSliwinski, TomaszHalterman, DennisŚliwka, MałgorzataHam, JungyeobSloan, DanielHamberg, LeenaSłomka, AnetaHambuckers, AlainSlusarenko, AlanHamerlynck, ErikSmeriglio, AntonellaHammond, JohnSmiley, RichardHampton, JohnSmith, AndrewHamrick, JamesSmith, KeithHan, DongWoonSmith, StanleyHan, KoeunSmolinska, BeataHan, YongSmułek, WojciechHanaka, AgnieszkaSobral, MarHanaoka, MitsumasaSobral, Rómulo S.Hanba, Yuko T.Socias, RafelHancock, Charles NathanSöderström, LarsHandoo, ZafarSoengas, Raquel G.Hanlon, MollySolanki, Manoj KumarHano, ChristopheSolano, Beatriz RamosHanslin, Hans MartinSolberg, SveinHansson, MatsSoldatenko, Sergei A.Hanus-Fajerska, EwaSolhaug, Knut AsbjørnHao, JianjunSoltész, AlexandraHardisson, ArturoSommerville, Karen DorothyHardy, KarenSon, Ki-HoHarman, GarySonah, HumiraHarmon, Carrie LapaireSong, GuoqingHarrington, Kerry C.Song, Jun-HoHart, RobbieSong, Yang-HeonHasanuzzaman, MirzaSonnewald, SophiaHase, YoshihiroSorce, CarloHasegawa, Paul MichaelSorgonà, AgostinoHasenstein, Karl HSorribas, F.J.Hashem, AbulSortino, GiuseppeHasiów-Jaroszewska, BeataSosnowska, KatarzynaHasnagas, Nikolaos D.Soteriou, GeorgiosHassan, Saad El-DinSoto, María JoséHassan, Sherif T. S.Sottile, FrancescoHassani-Pak, KeywanSõukand, RenataHasterok, RobertSoundararajan, PrabhakaranHaswell, Elizabeth S.Sousa Silva, MartaHatano, TsutomuSousa, Maria JoãoHatzilazarou, StefanosSouthwell, IanHauser, BernardSouzac, GustavoHavird, Justin CSowa, IreneuszHavrlentová, MichaelaSpanò, CarmelinaHavurinne, VesaSperdouli, IlektraHaworth, MatthewSpetz, Carl Jonas JorgeHawrył, MirosławSpiak, ZofiaHawrylak-Nowak, BarbaraSramko, GaborHay, FionaSreedasyam, AvinashHayes, Michael L.Srivastava, VibhaHazak, OraSrivedavyasasri, RadhakrishnanHe, JieSroka, ZbigniewHealey, AdamStacewicz-Sapuntzakis, MariaHeckmann, StefanŠtajner, NatašaHedden, PeterStana, Anca-DanielaHefferon, Kathleen L.Stanciu, OanaHegarty, MatthewStange, Claudia R.Hegazy, Mohamed-Elamir F.Staniak, MariolaHegedűsová, AlžbetaStankiewicz-Kosyl, MartaHeitz, ThierryStarfinger, UweHellevi Aalto, Sanni-LeeaStarkuviene, VytauteHelliwell, ChrisStarzak, KarolinaHellwig, FrankStasiak-Różańska, LidiaHeng, Lee KhengStaszak, Aleksandra MariaHenríquez, Jose LuisŞtefan, Daniela SiminaHenry, RobertStefanelli, DarioHenry, YvesŠtefanić, Petra PeharecHensel, GötzStefano, GiovanniHepworth, JoStefanou, StefanosHepworth, Shelley RStehlikova, DagmarHeras, JónathanSteiner, BarbaraHerde, MarcoStephenson, AndrewHeredia, AntonioStępień, ŁukaszHeredia-Guerrero, José AlejandroStępniowska, AnnaHéricourt, FrançoisSteup, MartinHermosa, RosaStevens, RebeccaHerrero, BaudilioStiburkova, BlankaHerschbach, CorneliaStobiecki, MaciejHesami, MohsenStodolak, BożenaHeuer, HolgerStoian, VladHey, StefanStojałowski, StefanHeyden, Hervé Van DerStoknes, KetilHeyl, AlexanderStoleru, VasileHibberd, JulianŠtolfa, IvnaHibrand-St.Oyant, LaurenceStout, Michael J.Hicks, LeslieStoyanova, AlbenaHigashi, YasuhiroStraumite, EvitaHiggs, DavidStrnad, MiroslavHigo, MasaoStrygina, KseniaHilhorst, Henk W. M.Strzałek, MałgorzataHill, CamillaStuart, Jeffrey J.Hilliou, FrederiqueStührwohldt, NilsHily, Jean-MichelStuper-Szablewska, KingaHinojosa, LeonardoSturtevant, DrewHinsinger, Damien DanielStütze, ThomasHirakawa, YoshihisaSu, DanHirata, TetsuyaSu, Huei-JiunHirata, YokoSubr, ZdenoHirel, BertrandSubramani, MayavanHirschi, KendalSubramanian, SowmyalakshmiHisano, HiroshiSudheeran, Pradeep KumarHjelmen, Carl E.Suetsugu, NoriyukiHleba, LukasSugier, PiotrHluska, TomášSugiyama, AkifumiHnatuszko-Konka, KatarzynaSujkowska-Rybkowska, MarzenaHnilicka, FrantisekSułkowska, MałgorzataHo, HonSumbwanyambe, MbuyuHo, Shin-LonSun, FengjieHo, Wing ShingSun, GenlouHoang, Nam V.Sun, MiaoHocevar, Barbara A.Sun, Tian-HuHodges, MichaelSung, JwakyungHodson, MartinSunkar, RamanjuluHoffman, Ava M.Sunseri, FrancescoHoffmann, Klaus H.Surapathrudu, KanakalaHoffmann, Lucia VieiraSurmacz, LilianaHofhansl, FlorianSurówka, EwaHofmann, RainerSuzuki, JonHöglind, MatsSuzuki, NobuhiroHohn, ThomasSuzuki-Yamamoto, ToshikoHojsgaard, DiegoSvartman, MartaHolalu, Srinidhi VŠvubová, RenátaHolb, ImreSwida-Barteczka, AleksandraHolland, CynthiaŚwieca, MichałHolloway, PaulSwiecilo, AgataHolst, NielsŚwierkosz, KrzysztofHolub, PetrSwiezewska, EwaHolubcik, MichalSwiezewski, SzymonHong, Chang PyoSykłowska-Baranek, KatarzynaHong, Chwan-YangSynowiec, AgnieszkaHong, YanSytar, OksanaHong, YiguoSytykiewicz, HubertHood, ElizabethSzakiel, AnnaHook, IngridSzalay, LaszloHooper, CorneliaSzczepanek, MałgorzataHopkins, Bryan G.Szekalska, MartaHorel, ÁgotaSzepesi, AgnesHorgan, FinbarrSzewczyk, AgnieszkaHori, KiyosumiSzewczyk, KatarzynaHoriguchi, GorouSzilvia, Tavaszi-SárosiHortigón-Vinagre, MaríaSzlachetko, Dariusz L.Horwitz, BenjaminSzmidt, Alfred EdwardHoshide, Aaron K.Szmyt, JanuszHowlett, BradSzopa, JanHsia, Shih-MinSzopka, KatarzynaHsiao, JerrySzparaga, AgnieszkaHsiao, VincentSztanke, MałgorzataHsieh, KuangwenSzulc, PiotrHsieh, Lu-ShengSzultka-Młyńska, MałgorzataHsu, Fu-ChiunSzumny, AntoniHu, HaixiaoSzweda, PiotrHu, ZhenbinSzwengiel, ArturHuang, Guan-JhongSzymanowska, UrszulaHuang, JianshengSzymańska, GrażynaHuang, JinlingSzymańska, Katarzyna PatrycjaHuang, JirongSzymańska, MagdalenaHuang, LeiSzymura, MagdalenaHuang, LinfangTabanca, NurhayatHuang, Meng-YuanTacchini, MassimoHuang, Nen-FuTada, YuichiHuang, Wen-LiiTadesse, MesfinHuang, Wu-JangTadesse, WuletawHuchzermeyer, BernhardTaguchi, YoshihiroHuesgen, PitterTaiki, MoriHughes, SamanthaTakabayashi, JunjiHunter, CharlesTakac, TomasHunter, Wayne BrianTakada, YoshinobuHuo, HeqiangTakahashi, HidekazuHura, TomaszTakahashi, MisaHúska, DaliborTakahashi, TakuHutter, MichaelTakahashi, ToshiyukiHwang, SeongbinTakakura, KohichiHyeonso, JiTakano, AtsukoHyodo, KiwamuTakasaki, HironoriHyskova, VeronikaTakenaka, ChisatoHyun, Tae KyungTakenaka, MizukiIannelli, Maria AdelaideTakeuchi, JunIantcheva, AneliaTalavera, MiguelIapichino, GiovanniTaleisnik, EdithIbáñez, JavierTalhinhas, PedroIbl, VerenaTalukder, ShyamalIbrahim, Mohamed AliTamás, LadislavIchim, Mihael CristinTamayo Martínez, ElisabethIchitani, KatsuyukiTamburino, RacheleIdrogo, Consuelo RojasTan, LiIelciu, IrinaTanase, CorneliuIentile, RiccardoTang, Gui-HuaIevinsh, GedertsTang, KaiIgamberdiev, Abir U.Tang, XiangruIglesias Fernández, RaquelTani, AkioIkeda, KenichiTannous, JoannaIkeda, YoshihisaTańska, MałgorzataIkeno, ShinyaTapia, Alejandro A.Ilieva, ZhenyaTarakanov, IvanImamura, SousukeTaranto, FrancescaImperatore, ConcettaTarasjev, AleksejImperiale, DavideTardella, Federico MariaInagaki, NoritoshiTarkowská, DanušeInbraj, StephenTarraf, WaedInfante, MartaTasso, BrunoIngelbrecht, IvanTava, AldoInnangi, MicheleTayeh, NadimInokuchi, TsutomuTchórzewska, DorotaInoue, AtsukoTegli, StefaniaInoue, HaruhikoTeh, Soon LiIoelovich, MichaelTeixeira, NatérciaIordache, VirgilTeixeira, RitaIorizzi, MariaTellgren-Roth, ChristianIqbal, MudassirTello, JavierIqdiam, BasheerTemu, VitalisIrakli, MariaTeng, ChongIrani, SolmazTeraishi, MasayoshiIrieda, HirokiTerentyev, Vasily V.Irving, LouisTermolino, PasqualeIsayenkov, StanislavTerpolilli, Jason JIshibashi, KazuhiroTerry, IreneIshijima, SumioTervahauta, Arja IIshikawa, RyujiTerzaghi, MattiaIslam, Md ShofiqTerzaghi, WilliamIslam, Md. TabibulTesic, ZivoslavIslam, Mohammad ZahirulTessema, Gezahegn GirmaItabashi, YutakaTesta, AntoninoIto, HisashiThakor, NehalIto, MichihoThalmann, MatthiasIto, YasuhiroThammina, Chandra SekharIturritxa, EugeniaThekkiniath, JoseIvanov, IvanThérèse Charles, MarieIvanov, RumenThéroux-Rancourt, GuillaumeIvanov, SergeyThien Nguyen, QuocIvanova, Donika G.Thirumalaikumar, Venkatesh P.Ivanova, LarissaThiruvengadam, MuthuIvetic, VladanThis, PatriceIvić, DarioThomas, Donald B.Iwase, AkiraThomas, JohnIzquierdo-Bueno, InmaculadaThomas, StefanJackson, Aaron K.Thomsen, Mette GoulJacobo-Velazquez, DanielThormann, ImkeJacygrad, EwelinaThurotte, AdrienJahnke, GizellaThussagunpanit, JutipornJaillais, YvonTian, JingkuiJain, Shri MohanTian, ShengJakob, TorstenTian, Shung WuJakovlevic, DraganaTintner, JohannesJakovljevic, MartinaTirado, Diego F.Jakubczyk, EwaTirado-Corbalá, RebeccaJakubska-Busse, AnnaTirillini, BrunoJallet, DenisTkacz, AndrzejJalli, MarjaTo, Kin-YingJames Hung, Keng-LouTobyn, GraemeJames, Euan KevinToda, YosukeJamnicka, GabrielaTodaka, DaisukeJan, Fuh-JyhTodeschini, ValeriaJanaćković, PedjaTodorova, DessislavaJanda, ElzbietaTofalo, RosannaJanda, KatarzynaTognetti, RobertoJaneczko, MonikaToiu, AncaJanga, MadhusudhanaTokarz, BarbaraJaniak, AgnieszkaTokatlidis, IoannisJanicka-Russak, MałgorzataTomassini, LambertoJaniszewska-Turak, EmiliaTomaszewski, DominikJanousek, BohuslavTomaz, IvanaJanovska, DagmarTombesi, SergioJanssen, DirkTomczyk, MichalJanuskaitiene, IrenaTomczykowa, MonikaJaqueth, JenniferTomi, FélixJaradat, AbdullahTomiczak, KarolinaJaradat, NidalTominaga, MotokiJarausch, BarbaraTomkowiak, AgnieszkaJarienė, ElvyraTomoiaga, Liliana LuciaJarocka-Karpowicz, IwonaTonelli, MicheleJaroszewicz, BogdanToriba, TaiyoJaroszewska, AnnaTorra, JoelJarret, RobertTorres, NazarethJarvis, DavidTorrini, GiuliaJarzebski, MaciejTorta, LivioJayawardena, Dileepa M.Toruńska-Sitarz, AnnaJeliazkova, EkaterinaToscano, StefaniaJemrić, TomislavToševski, IvoJenkins, ColinTóth, FerencJeon, Ji-HyeonTouzet, PascalJeong, Dong-HoonToyoda, YuJeong, Rae-DongToyota, KoikiJeong, Seon-YongTozawa, YuzuruJerkowić, IgorTran, TrongJewell, Jeremy B.Tranbarger, Timothy JohnJhanwar, ShaluTränkner, ConnyJi, LexiangTraveset, AnnaJiang, NanTravlos, IliasJiang, Shu-YeTraw, Milton BrianJiao, ShihuiTrdan, StanislavJighly, AbdulqaderTreder, KrzysztofJiménez Cantizano, AnaTres, Marcus ViniciusJiménez-Alfaro, BorjaTriantafyllou, IoannisJiménez-Monreal, Antonia M.Tribulato, AlessandroJin, ShuangxiaTrifilò, PatriziaJindo, KeijiTrigiano, RobertJo, Ick‑HyunTripodi, PasqualeJoachimiak, AndrzejTrocha, Lidia K.João Rodrigues, MariaTroia, AngeloJobe, TimothyTroitsky, AlexeyJócsák, IldikóTrolove, Stephen N.Johannes, WöstemeyerTrouvelot, SophieJohansson, EvaTrovato, MaurizioJohns, TimothyTroyo Diéguez, EnriqueJohnson, EricTrucillo, PaoloJohnson, Melissa A.Truniger, VerónicaJohnson, XenieTruzzi, FrancescaJokipii-Lukkari, SoileTsaballa, AphroditeJones, Gordon B.Tsai, Chen-ChiJones, JeffTsai, Yu-ChangJones, MattTsaniklidis, GeorgiosJones, Meriel G.Tsolakidou, Maria-DimitraJones, Michael B.Tsonev, TsonkoJones, Richard WTsouvaltzis, PavlosJordano, JuanTsyganov, Viktor E.Jordao, LuisaTucci, MarinaJorge, J. C. LuisTucker, Gordon C.Jougnot, DamienTucker, JamesJovanovic, ZivkoTudela, JoseJoyce, PriyaTufariello, MiriamJoyner, MatthewTuleja, MonikaJozwiak, AdamTullio, VivianJuárez, MiguelTullius Scotti, MarcusJuárez-Maldonado, AntonioTuncel, AytugJucan, DenisaTundis, RosaJukić, ŽeljkoTupys, AndriiJuliano, Claudia Clelia AssuntaTurkiewicz, Igor PiotrJullian, NathalieTurkovskaya, Olga V.Jung, Chol-HeeTusek, Ana JurinjakJung, Ki-HongTvrdá, EvaJung, Woo-JinTwardowska, IrenaJung, Yu-jinTyczewska, AgataJunkar, ItaTylova, EditaJuráček, MiroslavTyrka, MirosławJurado, M.M.Tyteca, DanielJurkonienė, SigitaTyunin, Alexey PJurkowska, RenataTzanova, MilenaJuzoń, KatarzynaTzortzakis, NikosKabir, Md. Shaha NurUcchesu, MarianoKabir, Mohammad FaujulUdrescu, LucretiaKačániová, MiroslavaUeda, AkihiroKacjan Maršič, NinaUeda, MinoruKaddes, AmineUesugi, AkaneKafuti, ChadrackUjházyová, MarianaKagan, Isabelle A.Ukken, FionaKagawa, NatsukoUlas, AbdullahKaitaniemi, PekkaUmber, MarieKalaji, Hazem MohamedUmehara, MikihisaKalemba, DanutaUnderwood, WilliamKalemba, Ewa M.Ungureanu, CameliaKalendar, RuslanUngurianu, AncaKaliniewicz, ZdzisławUpadhyay, RakeshKalisz, AndrzejUrban, OtmarKalivas, ApostolosUrbaniak, JacekKalle, RaivoUrlić, BranimirKalmbach, LotharUrminská, DanaKalogiouri, NatasaUrza, Alexandra K.Kałucka, IzabelaUsai, DonatellaKaminaka, HironoriUsami, ToshiyukiKamińska, IwonaUsenik, ValentinaKamionskaya, Anastasiya M.Usoltsev, Vladimir AndreevichKamiya, TakehiroUsuki, YoshinosukeKanamori, TakaoUttamani, JuhiKanavillil, NandakumarUygun, SahraKanayama, YoshinoriVaccino, PatriziaKandel, Devi RamVaibhav, KumarKanekatsu, MotokiVaid, NehaKang, Chang-KeunVaida, IoanaKang, HunseungVaknin, YiftachKang, Kwon-KyooValcke, RolandKangasjärvi, SaijaliisaValdés López, OswaldoKanto, TakeshiValdez-Aguilar, Luis AlonsoKappel, NoemiValentine, TracyKarady, MichalValenzuela, Juan LuisKarakashev, StoyanValera-Gran, DesiréeKarapanos, IoannisValkai, IldikóKaraś, MonikaValkov, EugeneKaražija, TomislavValkov, VladimirKarcz, DariuszVallès, JoanKargul, JoannaValletta, AlessioKarioti, AnastasiaVallon, OlivierKarkanis, AnestisVan De Wiel, Clemens M.Karlov, GennadyVan Deenen, NicoleKarppinen, KatjaVan Den Berg, CássioKarsaï, IldikóVan Den Broeck, LisaKarsznia, Krzysztof R.Van Den Ende, WimKashman, YoelVan Der Nest, Magriet AKasianov, Artem S.Van Doan, CongKasiotis, KonstantinosVan Straalen, Nico M.Kasiotis, KostasVan Tassel, DavidKašparovský, TomášVan Wuytswinkel, OlivierKassemeyer, Hanns-HeinzVan Zandt, PeterKaszycki, PawełVan, KyujungKatayama, AyumiVandebroek, InaKatsenios, NikolaosVanlerberghe, Greg C.Katsoyiannis, IoannisVantarová, KatarínaKatsuhara, MakiVányolós, AttilaKatz, OfirVarallyay, EvaKaundal, AmitaVarani, AlessandroKaushik, PrashantVargas-Murga, LilianaKaverin, Dmitry A.Vari, Camil-EugenKaviani, MinaVarone, LauraKavka, MareikeVarotto, SerenaKavroulakis, NektariosVasca Zamfir, DianaKawabe, AkiraVasenev, IvanKawade, KensukeVashisth, TriptiKawamura, FumioVasileva, VilianaKawaoka, AkiyoshiVassallo, AntonioKawashima, TomokazuVassilev, AndonKayama, MasazumiVassilev, NikolayKazuki, MotomuraVaverkova, MagdalenaKeasar, TamarVeatch-Blohm, MEKeasling, Jay D.Veen, Hans VanKeereetaweep, JantanaVek, ViljemKelliher, TimothyVelasco, LeonardoKellogg, Joshua J.Velena, AstridaKenfack, DavidVelikova, VioletaKępczyński, JanVella, Filomena MonicaKeren, SrđanVellak, KaiKern, JürgenVellios, EvangelosKesten, ChristopherVencill, WilliamKhaliluev, Marat R.Vendrell, JosepKhallouki, FaridVenezia, AccursioKhan, Mather AVenieraki, AnastasiaKhan, NaeemVenkatesh, JelliKhanal, SameerVerardo, VitoKhanh, TranVercesi, AlbertoKhapugin, Anatoliy A.Verdeja, Tamara HernándezKhaw, Kooi YepngVeres, SzilviaKhazaei, HamidVergine, MarziaKhosla, AashimaVerheggen, FrancoisKidoń, MarcinVernooy, RonnieKiefer, ChristianeVerstraeten, IngeKiefer, PatrickVezzulli, SilviaKiełbowicz-Matuk, AgnieszkaVicaș, Laura GrațielaKiełkowska, AgnieszkaVicent, AntonioKikowska, MałgorzataVicente, OscarKim, Cha YoungViczian, AndrasKim, Chang KilVidal-Meireles, AndréKim, ChoonsigVidican, RoxanaKim, DongjinVidigal, PatríciaKim, HyeranVidotto, FrancescoKim, Hyun-SoonViegas, WandaKim, In-JungVieira, Maria Lucia CarneiroKim, Jae-HyunVieira, PauloKim, JeongsikVielba, Jesús MaríaKim, Jin AVigani, GianpieroKim, Jin-KyungViketoft, MariaKim, Jung SunVikram, PrashantKim, Jung SungVilar, Miguel A.Kim, JunheonVilela, AliceKim, Ki HyunVilgis, ThomasKim, Kyung-MinViljevac Vuletić, MarijaKim, Min-SukVillano, CliziaKim, Yeon BokVillareal, Tracy A.Kim, Yong JuVinardell, José M.Kimbaris, AthanasiosVinceković, MarkoKimeklis, Anastasiia K.Vincent, DelphineKimura, SeisukeVincourt, PatrickKindlmann, PavelVincze, EvaKiniry, James R.Vining, KellyKirály, GergelyVirdi, KamaldeepKiraly, LorantVirgili, FabioKirker, Grant T.Viruel, MariaKiselev, Konstantin V.Viškelis, JonasKiseleva, AntoninaVita, FedericoKisková, TeréziaVitale, AlessandroKisnieriene, VilmaVitale, LucaKiss, RékaVitalini, SaraKitaoka, SatoshiVitamvas, JanKitchen, PhilipVítámvás, PavelKizis, DimosthenisVitasović Kosić, IvanaKlar, AgnesVitorino, Luciana CristinaKlčová, LenkaVitt, Dale H.Klein, Joshua D.Vitti, AntonellaKleintop, Adrienne E.Viuda-Martos, ManuelKlensporf-Pawlik, DorotaVives Cobo, CristinaKlepsch, Matthias MichaelVleugels, TimKleter, Gijs A.Vodeneev, VladimirKlikocka, HannaVodnar, Dan CristianKlíma, MiroslavVoigt, DagmarKlimek-Chodacka, MagdalenaVoigtmann, ThomasKlocko, Amy L.Vokurka, AlešKmieć, KatarzynaVolk, GayleKnickel, KarlheinzVolkov, VadimKnoll, JosephVoronkov, Alexander S.Knoop, VolkerVostinaru, OliviuKnox, OliverVráblová, MartinaKo, Dae KwanVu, Danh C.Ko, Jae-HeungVujcic, ValerijaKo, KisungVukovic, NinaKo, Swee-SuakVuts, JozsefKobae, YoshihiroVyhnánek, TomášKobayashi, KappeiVyrlas, PanagiotisKocábek, TomášWadas, WandaKocira, AnnaWade, RuthKocira, SławomirWaghmare, SakharamKocsy, GáborWaidmann, SaschaKocurek, MaciejWaligorski, PiotrKoczyk, GrzegorzWalker, Robert J.Kohyama, NorikoWalker, Robert P.Koide, Roger T.Walkowiak, SeanKoide, YoheiWallace, Joshua S.Koizumi, HiroyasuWallace, LisaKoizumi, TakahikoWallace, MichaelKojima, SoichiWalter, MonikaKokoska, LadislavWang, Chao-MinKoksharova, Olga A.Wang, DongmeiKolarcik, VladislavWang, JianboKolbert, ZsuzsannaWang, KanKollárová, KarinWang, NianKoller, RobertWang, PengKollers, SonjaWang, Richard R.-CKołodziejczyk-Czepas, JoannaWang, Sheng-YangKołton, AnnaWang, SishuoKolukisaoglu, ÜnerWang, Ting-fangKomenda, JosefWang, XuchuKomínek, PetrWang, YanbingKomives, TamasWang, YiKończak, MagdalenaWang, YingKong, LishengWang, YujieKonishi, MasaakiWang, ZhijunKono, MasaruWangchuk, PhurpaKonvalina, PetrWarchoł, MarzenaKopeć, AnetaWasowicz, PawelKopeć, PrzemysławWasternack, ClausKopsell, DavidWasterneys, GeoffreyKopta, TomášWaszczak, CezaryKoptur, SuzanneWatanabe, AtsushiKordalewska, MilenaWatanabe, Kazuo N.Korus, JarosławWatanabe, MakotoKorzeniowska, JolantaWatanabe, YuichiroKorzun, ViktorWegner, Lars H.Kosakowska, OlgaWehling, PeterKosakyan, AnushWehr, BernhardKoskela, ElliWei, RobertKosmala, ArkadiuszWei, XinghuaKosova, KlaraWeigt, DorotaKostova, DimitrinaWeil, Clifford FKoszel, MilanWeinhold, ArneKot, IzabelaWelsch, RalfKotilainen, TittaWendeborn, SebastianKottakota, ChandrasekharWeng, Mao-LunKou, YanjunWeng, XiaoyuKoubouris, GiorgosWenping, QiuKoudounas, KonstantinosWesołowski, MarekKougioumoutzis, KonstantinosWessler, SusanKougioumoutzis, KostasWeston, Leslie A.Koukounaras, AthanasiosWestphal, AndreasKourkoutas, IoannisWheeler, Terry AKováčik, JozefWhite, RosemaryKováčik, PeterWhitney, CoryKovács, Gábor M.Widemann, EmilieKovács, PéterWidrlechner, MarkKovinich, NikWieczfińska, JoannaKowalczyk, TomaszWieczorek, KingaKowalkowska, AgnieszkaWielbo, JerzyKowalska, JolantaWiesner-Reinhold, MelanieKowalski, RadosławWietrzyk-Pełka, PaulinaKozaki, AkikoWiland-Szymańska, JustynaKozieł, EdmundWilhelm, ChristianKozieradzka-Kiszkurno, MałgorzataWilke, OlafKozlov, Mikhail V.Willey, NeilKozłowska, MariolaWillick, IanKozma-Bognár, LászlóWillmund, FelixKraj, WojciechWilson, David C.Kramina, Tatiana E.Wimmer, Monika A.Krams, IndrikisWimmer, ZdenekKraska, ThorstenWin, Khin ThuzarKrasnyanski, SergeiWinefield, ChristopherKrasuska, UrszulaWink, MichaelKravchik, MichaelWinterhagen, PatrickKrawczyk, KatarzynaWintermantel, William M.Kreft, IvanWishart, JaneKregiel, Dorota MariaWitcher, AnthonyKrewenka, Kristin MarieWitte, Claus-PeterKrier, FrançoisWłodarczyk, MaciejKrigas, NikosWnorowski, ArturKrisch, JuditWojciechowski, KamilKrishna, Suresh Babu NaiduWójcik, Anna MariaKrishnamurthy, PannagaWójcik-Jagła, MagdalenaKrishnan, NatrajWójcikowska, BarbaraKrochmal-Marczak, BarbaraWojtunik-Kulesza, Karolina A.Krokidis, MariosWojtyla, ŁukaszKról, EwelinaWolfram, EvelynKropf, MatthiasWolverton, ChrisKroupin, PavelWon, So YounKrugman, TamarWone, BernieKrupa-Małkiewicz, MarcelinaWoodrow, PasqualinaKryštofová, SvetlanaWorthmann, AnnaKrzebietke, Sławomir JózefWoźniak, GabrielaKrzepiłko, AnnaWoznicki, Tomasz L.Krzyżak, JacekWright, Jessica W.Krzyzanek, VladislavWu, ChongmingKu, ChuanWu, HonghongKuan, Yu-HsiangWu, Li-HsinKubo, NakaoWu, ShengbiaoKubo, TomohikoWu, SonglinKučerová, AndreaWu, ZhiqiangKucharska, Alicja Z.Wyszkowski, MirosławKucharski, MariuszXanthopoulos, GeorgiosKućko, AgataXavier, Cesar Augusto DinizKucukoglu, MelisXavier, Nuno M.Kuderová, AlenaXia, QiongKudoyarova, GuzelXia, YeKudryavtsev, Denis S.Xiang, YuKukula-Koch, WirginiaXiao, JunKulhánek, MartinXiao, WenyanKulig, BogdanXin, ZhanguoKulik, AnnaXu, ChangjieKulik, TomaszXu, ChenpingKulkarni, DhananjayXu, ChunjianKull, TiiuXu, JuanKulma, AnnaXu, Kunning GabrielKulpa, DanutaXu, LeKulus, DariuszXu, PengKumar Kundu, JibanXu, XuanKumar, AmitXuan, Tran DangKumar, AnujXynias, Ioannis N.Kumar, JitendraYacoub, AmiraKumar, ManuYadav, Narendra SinghKumar, RatneshYadav, Vikash KumarKumar, RohitYakhin, OlegKumirska, JolantaYam, RitaKunjeti, Sridhara GYamaguchi, YubeKunji, EdmundYamane, KojiKunova, AndreaYamasaki, HideoKunwar, Ripu MARDHANYamauchi, TakakiKuo, Ping-ChungYan, HaidongKuo, Yen-WenYan, MaryKurczyńska, EwaYang, JinghuaKurek, MarcinYang, Rong-CaiKurihara, YukioYang, SeunghwanKuroda, KyoheiYang, WeibingKuronuma, TakanoriYang, WenyuKurowska, MarzenaYang, YuhengKurpaska, SławomirYaoita, YasunoriKurtti, TimothyYe, HengKurusu, TakamitsuYeh, Ting-fengKuś, PiotrYeung, ElaineKushunina, MariaYi, GibumKustrin, SnezanaYin, ChaoranKuta, ElżbietaYin, JingdongKuznetsov, Vladimir V.Yin, Nang HtayKuźniar, AgnieszkaYin, ShengKwak, SeonyeongYing, ShengKwasniewska, JolantaYing, TiejinKwiecien, IngaYockteng, Roxana B.Kwon, Choon-TakYokoi, ShujiKwon, Soon JaeYokota, ShinsoKwon, Soon WookYokoyama, RyusukeKyker, SarahYoneda, ReijiLabokas, JuozasYong, Jean W. H.Labouriau, RodrigoYoo, Ki-OugLabudda, MateuszYoo, MichelleLachowiec, JenniferYoshida, SatokoLacis, GunarsYoshida, TakuyaLacroix, BenoitYoshii, HidefumiLaghetti, GaetanoYou, ChenjiangLagunas, BeatrizYounès, DELLEROLahaye, MarcYruela, InmaculadaLaing, William AYu, JingyinLaitinen, Roosa A. E.Yu, Tien-ShinLamari, FotiniYu, XingwangLambardi, MaurizioYuan, HaiYingLambrev, PetarYuann, Jeu-Ming P.Lambreva, Maya D.Yusuke, KazamaLammers, Peter J.Zabot, Giovani L.Lamponi, StefaniaZagorchev, LyubenLan, PingZaina, GiusiLandi, LuciaZaini, PauloLandi, MarcoZaiter, AliLandi, SimoneZaitlin, DavidLandl, MagdalenaZakłos-Szyda, MalgorzataLandoni, MichelaZalabak, DavidLanfranco, LuisaZamborine-Nemeth, EvaLange, BastienZandstra, BernardLange, HolgerZannis, TheodorosLanger, Gitta JuttaZapata-Sierra, AntonioLangford, ZachZargar, MeisamLannacone, RinaZarrelli, ArmandoLanoue, JasonZarrouk, OlfaLantos, CsabaZarsky, ViktorLaranjo, MartaZarza, XavierLarkan, NicholasZavada, Michael S.Larkin, John C.Zbigniew, MiszalskiLarson, EmilyZdarta, AgataLarson, Steven R.Zdunek, ArturLaRue, ElizabethZdunic, GoranLastochkina, OksanaZeljkovic, CavarLászló Orbán, CsambalikZeljković, Sanja ĆavarLászló, MakraZellner, Wendy L.Latowski, DariuszZeng, LirongLatzel, VítZeng, Zhao-BangLau, Kin H.Zenta, NishioLauberts, MarisZerges, WilliamLaura, RossoZervoudakis, GeorgeLaval-Gilly, PhilippeŻesławska, EwaLavandera, Jose LuisZhang, AiLavin, MattZhang, BobLazarević, BorisZhang, ChunhuaLazarina, MariaZhang, DimingLeal, Ana MendesZhang, FeiLeal-Bertioli, SorayaZhang, HailinLeavitt, BryanZhang, JianhuiLebedev, VadimZhang, JunliLebedeva, Maria AlexandrovnaZhang, LiLebrun, ManhattanZhang, PengLech, KrzystofZhang, QingweiLedwożyw-Smoleń, IwonaZhang, ShibaoLee, Chang HoonZhang, WeiLee, HorimZhang, WenhengLee, In-ChulZhang, XiaoyuLee, Jeong-DongZhang, XuehuaLee, Jin-KooZhang, YuanmingLee, Jong HeeZhang, ZhenhuaLee, JunsooZhao, ChenchenLee, KwanukZhao, ChengLee, Kyeong-RyeolZhao, JinpingLee, Kyung JunZhao, LiyanLee, Sang JunZhao, MINGXIALee, Sang-HanZhao, TiebiaoLee, Soo InZhao, Xue QiangLee, Sung WooZheng, Xin-QiangLee, Tse-MinZhiponova, MiroslavaLee, Y.B.Zhong, ZhenhuiLee, YiZhou, BangjunLegaz González, María EstrellaZhou, MingqiLehnhoff, Erik A.Zhou, RongLehtonen, SamuliZhou, Wen-WenLei, ZhentianZhu, ChangfuLeidi, Eduardo O.Zhu, Qian-HaoLeisova-Svobodova, LeonaZhuang, XiaohongLeitão, Jorge H.Zhukov, Vladimir A.Leiva-Brondo, MiguelZhuo, ChunliuLejman, AgnieszkaŽiarovská, JanaLemaire, GillesZielenkiewicz, PiotrLemfack, Marie ChantalZielińska, SylwiaLengyel, AttilaZienkiewicz, AgnieszkaLenucci, Marcello SalvatoreZienkiewicz, KrzysztofLeollini, LuisaZingaretti, Sonia MarliLeón-Rivera, IsmaelZiogas, VasileiosLeontina, LipanZivanovic, JasminaLepedus, HrvojeZłotek, UrszulaLeporini, MariarosariaZou, CengLeshem, YehoramZou, JunjieLesueur, DidierZou, ZhongweiLevi, AmnonZovko Končić, MarijanaLewinska, AnnaZsuzsanna, PluhárLexa, MatejZub, KarolLey, Alexandra C.Zuber, HélèneLeyva Pérez, Maria De La OZucca, PaoloLi, Fu-shuangŻuk, MagdalenaLi, GangZuzarte, MónicaLi, LingŽvingila, DonatasLi, Po-HsienZwart, RebeccaLi, Qian-ShengZweifel, RomanLi, QiongyuZwiazek, JanuszLi, ShaoZygadlo, JulioLi, WanlongŻyżelewicz, DorotaLi, Xiangnan


